# Global trends in antimicrobial resistance of *Enterococcus faecium*: a systematic review and meta-analysis of clinical isolates

**DOI:** 10.3389/fphar.2025.1505674

**Published:** 2025-04-07

**Authors:** Chen Huang, Samaneh Moradi, Mohammad Sholeh, Faezeh Motallebi Tabaei, Tingting Lai, Bo Tan, Jingjing Meng, Khalil Azizian

**Affiliations:** ^1^ Research Centre of Basic Intergrative Medicine, School of Basic Medical Sciences, Guangzhou University of Chinese Medicine, Guangzhou, Guangdong, China; ^2^ Department of Microbiology, Faculty of Medicine, Golestan University of Medical Sciences, Gorgan, Iran; ^3^ Department of Bacteriology, Pasteur Institute of Iran, Tehran, Iran; ^4^ College of Health, Binzhou Polytechnic, Binzhou, Shandong, China; ^5^ Department of Microbiology, Faculty of Medicine, Kurdistan University of Medical Sciences, Sanandaj, Iran; ^6^ Zoonoses Research Center, Research Institute for Health Development, Kurdistan University of Medical Sciences, Sanandaj, Iran

**Keywords:** antimicrobial resistance, *Enterococcus faecium*, systematic review and meta-analysis, vancomycin, clinical isolate bacterial

## Abstract

**Background:**

Multidrug-resistant bacteria are associated with a high number of deaths and pose a significant global concern. In recent decades, among these resistant bacteria, *Enterococcus faecium*, a hospital-acquired pathogen, has attracted more attention.

**Objective:**

The present study aims to document the current state of resistance in *E. faecium* globally by considering several variables, including geographical locations, temporal trends, and sources of infection.

**Methods:**

We searched studies in PubMed, Scopus, and Web of Science (30 November 2022). All statistical analyses were carried out using the statistical package R.

**Results:**

Our meta-analysis of antibiotic resistance across various clinical isolates revealed substantial heterogeneity and variability. The average resistance proportions ranged from 2% for linezolid to 62.8% for erythromycin, with significant differences observed across different time periods, countries, and World Health Organization regional offices.

**Conclusion:**

Our findings confirm the high antibacterial activity of linezolid against *E. faecium* isolates. Additionally, our investigation reveals a gradual increase and a concerning upward trend in resistance rates for nearly all agents in recent years. However, the significant reduction in resistance rates for certain antibiotics suggests that these drugs could potentially regain their effectiveness in the future.

## Introduction

Multidrug-resistant (MDR) bacteria have emerged as a serious global public health threat (Algammal A et al., 2023). These MDR bacteria are associated with a high number of deaths and contribute to increased costs for both patients and the healthcare system (Kilbas and Ciftci, 2018). In recent decades, among these resistant bacteria, *Enterococcus* spp. has attracted more attention (Bhardwaj, 2019; Fiore et al., 2019). *Enterococci* are Gram-positive cocci, facultative anaerobic bacteria commonly found in humans and animals and known as dominant gastrointestinal flora (Kim and Koo, 2020; Lee et al., 2021). Although *Enterococcus* species are part of the human microbiota, certain species have emerged as significant pathogens in recent decades, particularly among hospitalized and immunocompromised patients (Výrostková et al., 2021). *Enterococcus faecalis* and *Enterococcus faecium* account for over 90% of *Enterococcus* isolates recovered from human patients (Kilbas and Ciftci, 2018; Woźniak-Biel et al., 2019).

*E. faecium* is a major nosocomial pathogen responsible for a variety of infections, including bloodstream infections, urinary tract infections (UTIs), and endocarditis, particularly in immunocompromised and hospitalized patients (Jahansepas et al., 2018; Gorrie et al., 2019). Its ability to colonize the gastrointestinal tract makes *E. faecium* a significant reservoir for infection, with studies estimating colonization rates in hospitalized patients ranging from 10% to 40% (Saifi et al., 2008; Pidot et al., 2018; Mirzaii et al., 2023). However, the global incidence of colonization compared to infection remains underreported, highlighting the need for comprehensive epidemiological studies (Bhatt et al., 2015; Emaneini et al., 2016; Melese et al., 2020).

A significant proportion of UTIs in hospitalized patients are caused by *E. faecium*, particularly in patients with risk factors such as indwelling catheters, underlying comorbidities, or prior antibiotic use (Codelia-Anjum et al., 2023). Codelia-Anjum et al. (2023) and Codelia-Anjum et al. (2023) highlighted the growing burden of enterococcal UTIs and their resistance to commonly used antibiotics, posing challenges for effective treatment. In addition to UTIs, *E. faecium* is a notable cause of infective endocarditis, especially in patients with prosthetic heart valves or a history of invasive cardiac procedures (Babeș et al., 2021). and Babeș et al. (2021) described the severe clinical outcomes of infective endocarditis caused by *E. faecium*, which often requires combination antibiotic therapy and is associated with high morbidity and mortality risks. Beyond these, *E. faecium* contributes to bloodstream infections (BSIs), intra-abdominal infections, surgical site infections, pelvic infections, and wound infections, particularly in hospitalized or immunocompromised patients (Arias and Murray, 2012; Sangiorgio et al., 2024). Its ability to colonize the gastrointestinal tract often precedes these infections, serving as a reservoir and entry point for systemic disease, particularly under conditions of antibiotic pressure and compromised immunity (Donskey, 2004; Arias and Murray, 2012). These diverse infection types underscore the importance of targeted surveillance and appropriate antimicrobial strategies to manage *E. faecium*-related diseases in both hospital and community settings. While *E. faecium* was previously recognized primarily as a pathogen in hospitalized patients, recent reports indicate a significant increase in its role in community-acquired infections (Agus et al., 2006).

In addition to its role in various infections, the inherent and acquired resistance of *E. faecium* is an important consideration. Generally, this bacterium can acquire resistance through different mechanisms, including gene mutations and gene transfer from other bacteria (Mirzaii et al., 2023). Gene transfer in this bacterium is one of the main reasons for antimicrobial resistance among bacteria. The development of resistance is associated with decreased antimicrobial effectiveness and increased morbidity and mortality (Kim and Koo, 2020). In the 1970s, increased resistance to third-generation cephalosporins and ampicillin was observed. Due to the increased resistance rate, vancomycin is recommended as the first option for the treatment of infection caused by enterococci. However, in 1986, resistance to vancomycin was reported in the United Kingdom and France (Emaneini et al., 2016; Jubeh et al., 2020). *E. faecium* has garnered significant attention due to its resistance to vancomycin, one of the last-resort antibiotics for treating severe Gram-positive infections (Ahmed Mo and Baptiste, 2018). This resistance poses a critical challenge in treating infections caused by this pathogen, particularly in healthcare settings. Consequently, vancomycin-resistant *E. faecium* (VRE) has been listed by the World Health Organization (WHO) as a priority pathogen for which the development of new treatment strategies is essential (Asokan et al., 2019). Because infections caused by VRE are associated with more hospitalization, generating enormous costs and increased mortality (Melese et al., 2020), knowledge about control and treatment strategies is necessary.

Previous systematic reviews have investigated resistance in enterococci; however, all studies primarily reported local resistance, vancomycin resistance, or strains of enterococci that were resistant and recovered from specific infections (Emaneini et al., 2016; Kilbas and Ciftci, 2018; Melese et al., 2020; Correa-Martínez et al., 2021; Yan et al., 2023). To our knowledge, neither statistical analysis nor a comprehensive assessment of enterococci resistance in all infections was conducted in those meta-analyses. Therefore, in the present study, we focused on documenting the current state of resistance in *E. faecium* by analyzing relevant literature published worldwide. Also, we included several variables such as geographical locations, time trends, and sources of infection in the analysis.

## Methods

This review is reported in accordance with the Preferred Reporting Items for Systematic Reviews and Meta-Analyses guidelines (PRISMA) (Moher et al., 2009).

### Search strategy and study selection

We systematically searched for relevant articles in PubMed, Scopus, and Embase (Until 30 November 2022) by using the related keywords: (“Enterococci” OR “Enterococcus faecium” OR “E. faecium” AND “antimicrobial” OR “antibiotic” AND “resistance” OR “susceptible” OR “susceptibility” OR “minimum inhibitory concentration” OR “MIC”) in the title/abstract/keywords fields. No limitation was used while searching databases. The search strategy was designed and conducted by the investigators of the study. The reference lists of all related studies were reviewed for additional publications. The records obtained through database searches were merged, and duplicates were removed using EndNote X8 (Thomson Reuters, New York, NY, United States). One of the team members randomly evaluated the search results to ensure that no relevant studies were overlooked. The authors collaborated on all steps, resolving any disagreements about article selection through discussion. Additionally, references from the reviewed articles were also searched for further information.

### Inclusion and exclusion criteria

The eligibility criteria for including articles in the meta-analysis were as follows: 1) original studies investigating antibiotic resistance in *E. faecium* isolates collected exclusively from human clinical samples; 2) peer-reviewed articles published in English between 2000 and 2022; 3) studies that specified the total number of *E. faecium* clinical isolates; and 4) studies that specified the number of antibiotic-resistant *E. faecium* clinical isolates. The exclusion criteria were as follows: 1) studies that contained duplicate data or were overlapping; 2) studies without clinical isolates; 3) studies reporting antibiotic resistance of *Enterococcus* species other than *E. faecium*; 4) reviews, cohort studies, pharmacokinetic studies, and conference abstracts; 5) studies in which antibiotic resistance rates were not clearly presented or reported; 6) studies that included clinical samples from animals or the environment (i.e., related to the One Health concept).

## Data extraction

The following information was extracted from each included study: first author, publication year, continent, country, number of *E. faecium* clinical isolates, number of antibiotic-resistant *E. faecium* clinical isolates, infection source (bloodstream, gastrointestinal tract, urinary tract, or mixed), and antimicrobial susceptibility testing (AST) methods (MIC-based methods and disk diffusion agar). Data were collected by two independent reviewers and verified by a third researcher. The resistance rate was calculated as the number of resistant isolates divided by the total number of isolates tested.

### Quality assessment

The quality of the included studies was independently assessed by two reviewers using an adapted version of the Newcastle–Ottawa scale. This adaptation was specifically tailored for cross-sectional studies to evaluate key factors such as selection, comparability, and outcome assessment in the context of observational research (Modesti et al., 2016). A score ranging from 0 to 8 points was attributed to each study (≥6 points: high quality, ≤5 points: low quality). A higher score indicated a higher study quality. A third reviewer was assigned (or adjudicated) in any cases of disagreement.

### Statistical analysis

The studies presenting raw data on antibiotic resistance in *E. faecium* clinical isolates derived from humans were included in the meta-analysis that was carried out using the meta-prop (Schwarzer, 2007) command in R statistical software on all prevalence statistics by antibiotic, region (continents/countries), year, infection source, and AST. The meta-analysis results consist of a prevalence statistic with 95% confidence intervals calculated from the weighted prevalence statistics for all the studies in the specified subgroup by antibiotic, region (continents/countries), year, infection source, and AST. Publication bias was assessed using Egger’s test. All statistical interpretations were reported on a 95% confidence interval (CI) basis. All statistical analyses were carried out using the statistical package R 3.6.0 (R Foundation for Statistical Computing: Vienna, Austria) (Team, 2013).

### Study outcomes

Resistance data were interpreted according to the Clinical and Laboratory Standards Institute (CLSI) Weinstein (2020) and The European Committee on Antimicrobial Susceptibility Testing (2025) (EUCAST) guidelines as stated in the included studies. Subgroup analyses were performed based on the following categories: 1) year (2000–2019, 2020–2022), 2) geographical area (continents/countries), 3) infection source, 4) interpretation standards (CLSI and EUCAST), and 5) AST methods.

## Results

### Systematic literature search

**FIGURE 1:**
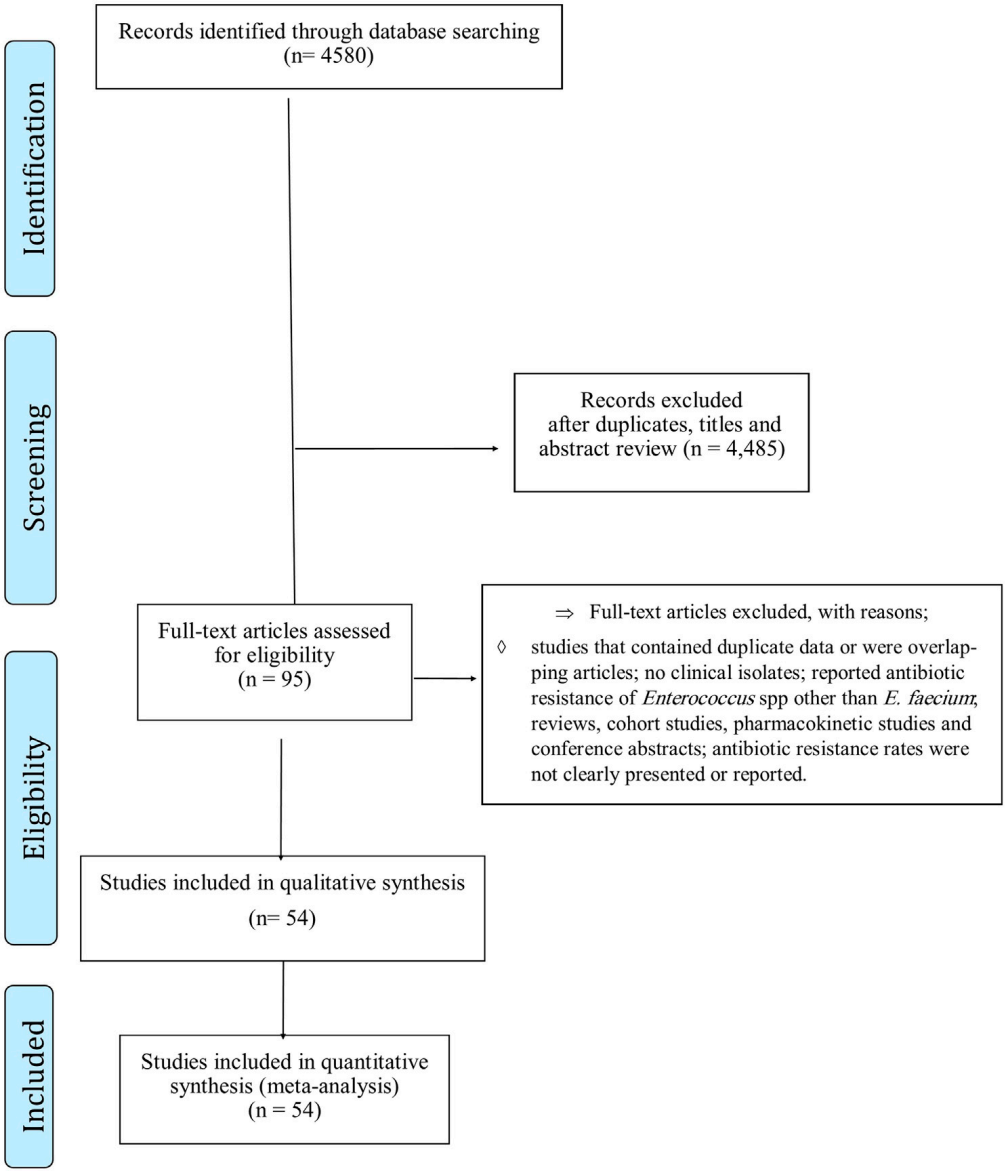
PRISMA flowchart of included studies.

A total of 4,580 records were identified in the initial search. After screening the titles and abstracts, 4,485 articles were excluded due to irrelevance and duplication. The full texts of the remaining 95 articles were then reviewed (Figure 1), and 41 were further excluded for the aforementioned reasons. Finally, the 54 studies included (Luh et al., 2000; Zouain and Araj, 2001; Richter et al., 2003; Udo et al., 2003; Boost et al., 2004; Karmarkar et al., 2004; Brauers et al., 2005; Hsueh et al., 2005; Kaçmaz and Aksoy, 2005; Kapoor et al., 2005; Oh et al., 2005; Nicoletti et al., 2006; Quiñones-Pérez et al., 2006; Ghanem et al., 2007; Sader et al., 2007; Saifi et al., 2008; Sader and Jones, 2009; Jain et al., 2011; Liu et al., 2011; Olawale et al., 2011; Batistão et al., 2012; Djahmi et al., 2012; Dworniczek et al., 2012; Kelesidis et al., 2012; Pourakbari et al., 2012; Sibel et al., 2012; Balaei Gajan et al., 2013; Dadfarma et al., 2013; Fernandes and Dhanashree, 2013; Kafil et al., 2013; Lee et al., 2013; Jia et al., 2014; Bhatt et al., 2015; Li et al., 2015; Jahansepas et al., 2018; Haghi et al., 2019; Zhang et al., 2019; Condò et al., 2020; Dave et al., 2020; Davis et al., 2020; Dodson et al., 2020; Dong et al., 2020; Erdem et al., 2020; Franyó et al., 2020; Friedman et al., 2020; Guo et al., 2020; Jannati et al., 2020; Rostkowska et al., 2020; Tollu and Ekin, 2020; Wang et al., 2020; Arbune et al., 2021; Boccella et al., 2021; Bogut et al., 2021; Coombs et al., 2022) were published between 2000 and 2022 (Supplementary File S1). The screening and selection process are summarized in the PRISMA flow chart (Figure 1).

### Characteristics of included studies

**FIGURE 2:**
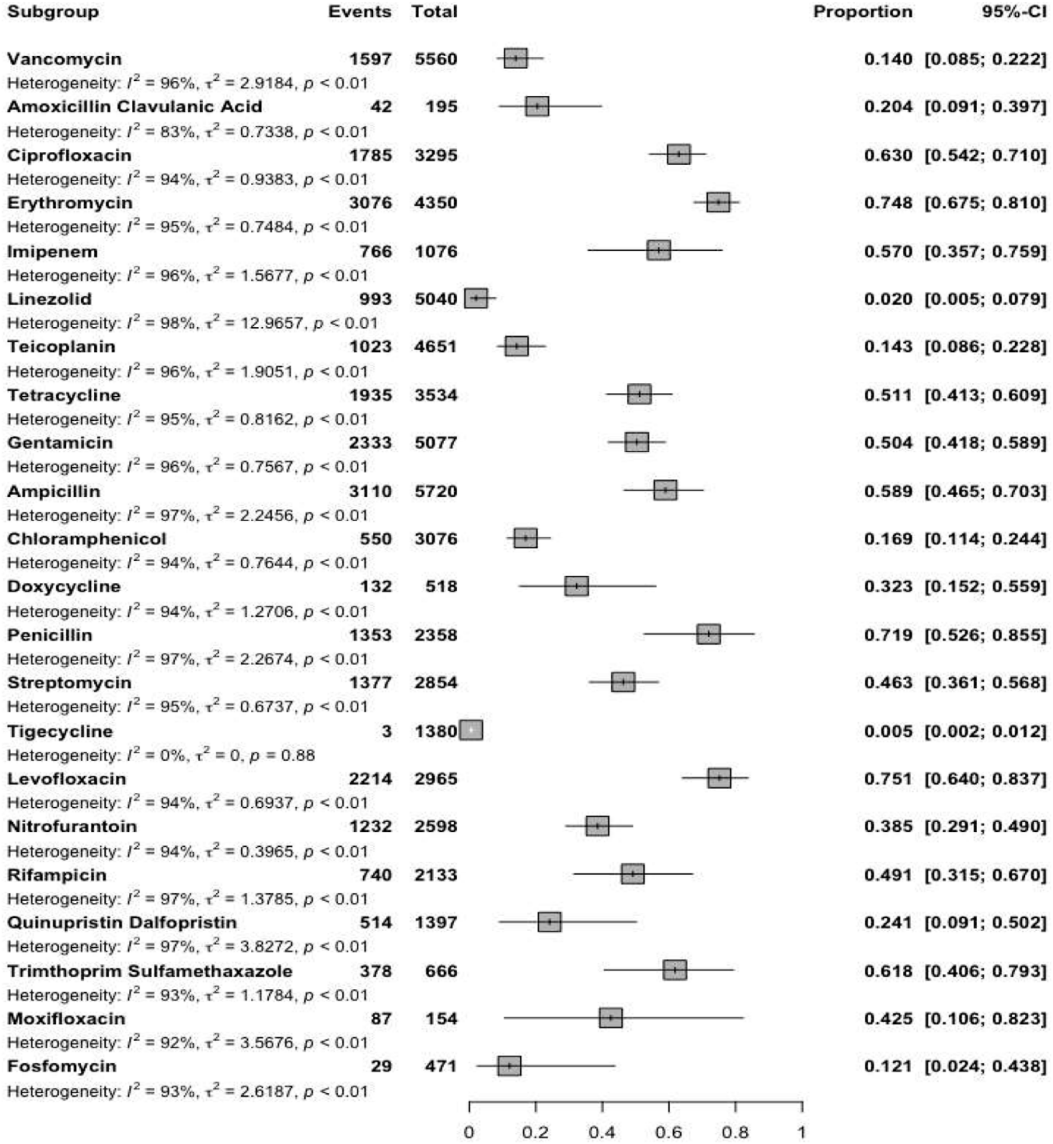
Forrest plots of included antibiotics.

**FIGURE 3:**
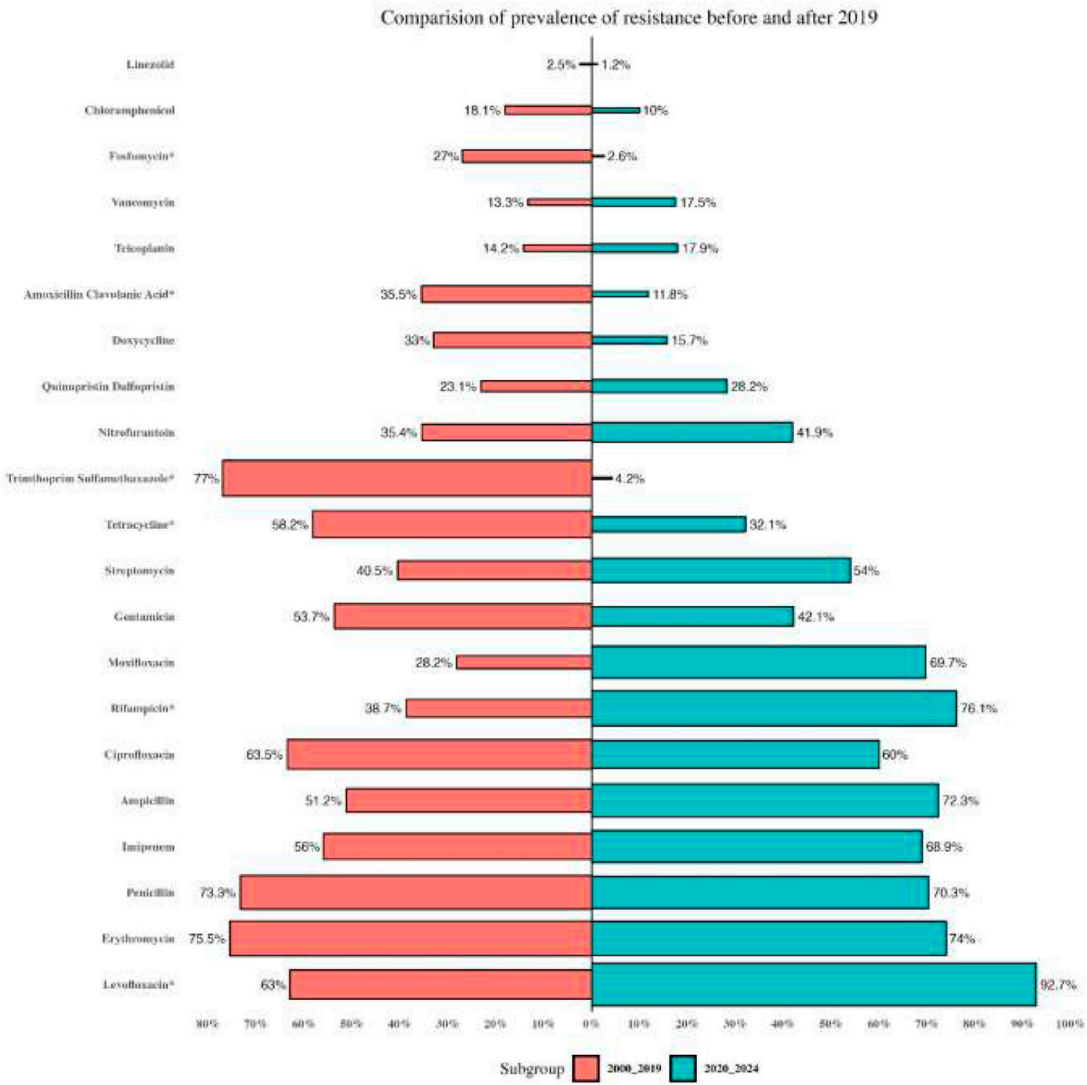
The changes in the proportion of antibiotic resistance over time.

**FIGURE 4:**
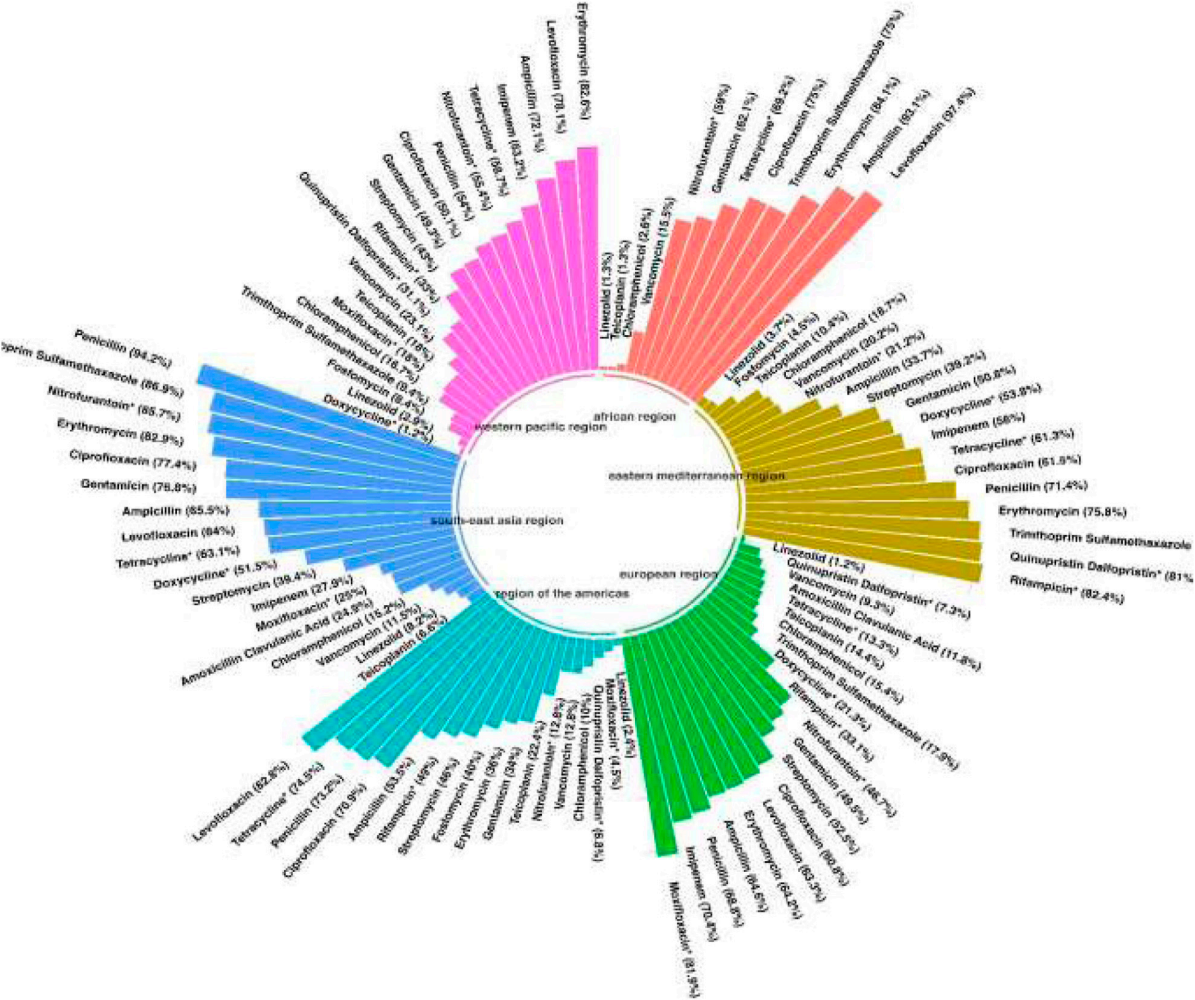
The changes in the proportion of antibiotic resistance based on WHO regions.

Reports were collected from 21 countries across five continents: Europe (Poland, Hungary, Italy, Germany, Romania), Asia (Iran, Turkey, China, South Korea, Kuwait, Israel, Taiwan, India, Lebanon, and Hong Kong), the Americas (United States, Brazil, Cuba), Oceania (Australia), and Africa (Algeria and Nigeria). Figure 2 presents forest plots of the proportions of resistant isolates to selected antibiotics. The proportions for each antibiotic and subgroup analyses by continent/country, genus, species, and AST method are detailed in Supplementary File S2. Temporal changes in resistance proportions to selected antimicrobials are displayed in Figure 3. Figure 4 illustrates the changes in resistance proportions by WHO regions for selected antibiotics. The trends in resistance rates are summarized below.

### Vancomycin

A total of 5,560 clinical isolates from 46 studies were included in the analysis of vancomycin resistance. The estimated average proportion using a random-effects model was 14% (95% CI: 8.5–22.2), with substantial heterogeneity observed between studies (I^2^ = 96.45%, P < 0.001) (Supplementary File S2; Figure 2). A subgroup analysis was performed for two periods: 2000–2019 and 2020–2022 (Supplementary File S2) to examine trends over time. Vancomycin resistance increased from 13.3% (95% CI: 6.4–25.4) during 2000–2019 to 17.5% (95% CI: 9.4–30.3) in 2020–2022 (Supplementary File S2; Figure 3; P = 0.741). Among the 20 countries reporting vancomycin resistance, 13 countries (Poland, Iran, China, United States, Brazil, Cuba, South Korea, Hungary, Israel, Australia, Taiwan, India, and Nigeria) reported resistance rates above 5%. A statistically significant difference was observed in resistance rates between countries (P < 0.001). The highest resistance proportion was recorded in the western Pacific region, at 23.1%, whereas the lowest was in Europe, at 9.3% (Figure 4). Urinary tract infections showed the highest resistance proportion, at 37.4% (95% CI: 22.8–54.8), while mixed infections had the lowest, at 7.1% (95% CI: 2.6–18) (Supplementary File S2).

### Teicoplanin

A total of 4,651 clinical isolates from 30 studies were included in the analysis of teicoplanin resistance. The estimated average proportion using a random-effects model was 14.3% (95% CI: 8.6–22.8), with substantial heterogeneity observed between studies (I^2^ = 95.7%, P < 0.001) (Supplementary File S2; Figure 2). Teicoplanin resistance gradually increased from 14.2% (95% CI: 7.9–24.2) in 2000–2019 to 17.9% (95% CI: 5.5–45.2) in 2020–2022 (Supplementary File S2; Figure 3; P = 0.619). Among 16 countries reporting teicoplanin resistance data, 10 countries (Iran, Turkey, Cuba, Hungary, Poland, China, Australia, United States, Taiwan, and India) reported resistance rates above 5%. The highest resistance proportion was observed in the Americas, at 22.4%, while the lowest was in Africa, at 1.3% (Figure 4). BSIs showed the highest resistance proportion, at 20.2% (95% CI: 4.5–57.6), whereas gastrointestinal tract infections had the lowest, at 4.9% (95% CI: 0–95.2) (Supplementary File S2).

### Penicillin

A total of 2,358 clinical isolates investigated in 16 studies were included in the analysis of penicillin resistance. The estimated average proportion based on the random-effects model was 71.9% (95% CI, 52.6, 85.5) with substantial heterogeneity (I^2^ = 97.14%%, *P*= <0.001) observed between included studies (Supplementary File S2; Figure 2). In the case of penicillin, we found a minor decrease in the percentage of resistance between the two time periods as follows: from 73.3% (95% CI 39.4–82.3) resistance among 972 strains in 2000–2019 to 70.3% (95% CI 38.6–89.9) resistance among 381 strains in 2020–2022. (Supplementary File S2; Figure 3; P = 0.843). Among eight countries reporting resistance data for penicillin, six (Iran, Turkey, China, Brazil, United States, Australia, Taiwan, and India) reported that >70% of isolates had penicillin resistance. There was a statistically significant difference in the penicillin resistance rates between countries (P < 0.001). The highest recorded proportion of resistance, 94.2%, was observed in Southeast Asia, while the western Pacific region reported the lowest, 54% (Figure 4). The subgroup analysis conducted on penicillin resistance based on infection source revealed a significant difference in resistance proportions (P *=* 0.002).

### Ampicillin

A total of 5,720 clinical isolates investigated in 40 studies were included in the analysis of ampicillin resistance. The estimated average proportion based on the random-effects model was 58.9% (95% CI, 46.5, 70.3), with substantial heterogeneity (I^2^ = 97.43%, *P* = <0.001) observed between included studies (Supplementary File S2; Figure 2). The proportion of ampicillin resistance gradually increased from 51.2% (95% CI 38.2–64) of 3,585 strains in 2000–2019 to 72.3% (95% CI 50.9–86.8) of 2,135 strains in 2020–2022 (Supplementary File S2; Figure 3; P = 0.083). Among 17 countries reporting resistance data for ampicillin, only four countries (South Korea, Italy, and Lebanon) reported that <25% of isolates had ampicillin resistance. There was a statistically significant difference in the ampicillin resistance rates between countries (P *=* 0.03). The highest recorded proportion of resistance was observed in the African region, at 93.1%, while the eastern Mediterranean region reported the lowest recorded proportion, reaching 33.7% (Figure 4). The subgroup analysis conducted on ampicillin resistance based on infection source observed a significant difference in resistance proportions. Urinary tract infections reported the highest proportion of resistance, at 60.7% (95% CI 17.7–91.7), while gastrointestinal tract infections reported the lowest proportion, at 27.8% (95% CI 1.6–90.1) (Supplementary File S2).

### Amoxicillin–clavulanic acid

A total of 195 clinical isolates investigated in four studies were included in the analysis of amoxicillin-clavulanic acid resistance. The estimated average proportion based on the random-effects model was 20.4% (95% CI, 9.1, 39.7), with substantial heterogeneity (I^2^ = 83.14%, *P* < 0.001) observed between included studies (Supplementary File S2; Figure 2).

### Imipenem

A total of 1,076 clinical isolates investigated in nine studies were included in the analysis of imipenem resistance. The estimated average proportion based on the random-effects model was 57% (95% CI, 35.7, 75.9), with substantial heterogeneity (I^2^ = 96.08%, *P* < 0.001) observed between included studies (Supplementary File S2; Figure 2).

### Gentamicin

A total of 5,077 clinical isolates investigated in 31 studies were included in the analysis of gentamicin resistance. The estimated average proportion based on the random-effects model was 50.4% (95% CI, 41.8, 58.9), with substantial heterogeneity (I^2^ = 95.86%, *P* < 0.001) observed between included studies (Supplementary File S2; Figure 2). The proportion of gentamicin resistance gradually decreased from 53.7% (95% CI 43–64) of 1,683 strains in 2000–2019 to 42.1% (95% CI 26.8–59.2) of 650 strains in 2020–2022 (Supplementary File S2; Figure 2; P = 0.262). Among 15 countries reporting resistance data for gentamicin, 13 (Poland, Iran, Turkey, Cuba, United States, Italy, Australia, Taiwan, India, Hong Kong, Algeria, Nigeria, and South Korea) reported that >25% of isolates had gentamicin resistance. The highest recorded proportion of resistance was observed in Southeast Asia, at 76.8%, while the region of the Americas reported the lowest, reaching 34% (Figure 4). Urinary tract infections reported the highest proportion of resistance, at 70.7% (95% CI 53.5–83.5), while gastrointestinal infections reported the lowest proportion, at 37.1% (95% CI 22.9–54) (Supplementary File S2).

### Streptomycin

A total of 2,854 clinical isolates investigated in 18 studies were included in the analysis of streptomycin resistance. The estimated average proportion based on the random-effects model was 46.3% (95% CI, 36.1, 56.8), with substantial heterogeneity (I^2^ = 95.30%, *P* < 0.001) observed between included studies (Supplementary File S2; Figure 2). The proportion of streptomycin resistance gradually increased from 40.5% (95% CI 31.3–50.4) in 2000–2019 to 54% (95% CI 33.4–73.4) in 2020–2022 (Supplementary File S2; Figure 3; P = 0.080). There was no significant difference in the streptomycin resistance rates between countries (P = 0.882). The highest recorded proportion of resistance was observed in the European region, at 52.5%, while the eastern Mediterranean region reported the lowest recorded proportion, reaching 39.2% (Figure 4). Urinary tract infections reported the highest proportion of resistance, at 71.2% (95% CI 34.4–92.1), while BSIs reported the lowest proportion, at 33.2% (95% CI 28.3–38.5) (Supplementary File S2).

### Ciprofloxacin

A total of 3,295 clinical isolates investigated in 34 studies were included in the analysis of ciprofloxacin resistance. The estimated average proportion based on the random-effects model was 63% (95% CI, 55.1, 71.8), with substantial heterogeneity (I^2^ = 94.21%, *P* < 0.001) observed between included studies (Supplementary File S2; Figure 2). The proportion of ciprofloxacin resistance showed a minor decrease, changing from 63.5% (95% CI 52.2–73.5) in 2000–2019 to 60% (95% CI 46.2–78.1) in 2020–2022 (Supplementary File S2; Figure 3; P *=* 0.957). Among 16 countries reporting resistance data for ciprofloxacin, nine countries (China, Brazil, South Korea, Turkey, United States, Australia, India, Hong Kong, and Nigeria) reported that >45% of isolates had ciprofloxacin resistance. The highest recorded proportion of resistance was observed in Southeast Asia, exhibiting a proportion of 77.4%, while the western Pacific region reported the lowest recorded proportion of 50.1% (Figure 4). BSIs reported the highest proportion of resistance, at 60.1%, while gastrointestinal tract infections reported the lowest proportion, at 39.1% (Supplementary File S2).

### Levofloxacin

A total of 2,965 clinical isolates investigated in 13 studies were included in the analysis of ciprofloxacin resistance. The estimated average proportion based on the random-effects model was 75.1% (95% CI, 64, 83.7) with substantial heterogeneity (I^2^ = 94.40%, *P* < 0.001) observed between included studies (Supplementary File S2; Figure 2). The proportion of ciprofloxacin resistance significantly increased from 63% (95% CI 53.5–71.7) in 2000–2019 to 92.7% (95% CI 85.2–96.6) in 2020–2022 (Supplementary File S2; Figure 3; P < 0.001). Among seven countries reporting resistance data for ciprofloxacin, six (China, United States, Germany, India, Hong Kong, and Algeria) reported that >45% of isolates had ciprofloxacin resistance. The highest recorded proportion of resistance was observed in the African region, exhibiting 97.4%, while Southeast Asia reported the lowest recorded proportion of 64% (Figure 4). A significant difference was found in the AST method (P = 0.018).

### Moxifloxacin

A total of 154 clinical isolates investigated in five studies were included in the analysis of moxifloxacin resistance. The estimated average proportion based on the random-effects model was 42.5% (95% CI, 10.6, 82.3) with substantial heterogeneity (I^2^ = 92.50%, *P* < 0.001) observed between included studies (Supplementary File S2; Figure 2).

### Tigecycline

A total of 1,380 clinical isolates investigated in seven studies were included in the analysis of tigecycline resistance. The estimated average proportion based on the random-effects model was 0.5% (95% CI, 0.2, 1.2) (Supplementary File S2; Figure 2).

### Tetracycline

A total of 3,534 clinical isolates investigated in 18 studies were included in the analysis of tetracycline resistance. The estimated average proportion based on the random-effects model was 51.1% (95% CI, 41.3, 60.9), with substantial heterogeneity (I^2^ = 94.80%, *P* < 0.001) observed between included studies (Supplementary File S2; Figure 2). The proportion of tetracycline resistance decreased from 58.2% (95% CI 47.8–67.9) in 2000–2019 to 32.1% (95% CI 14.3–57.3) in 2020–2022 (Supplementary File S2; Figure 3; P *=* 0.016). There was a significant difference in the tetracycline resistance rates between countries (P = 0.003). The highest recorded proportion of resistance was observed in the region of the Americas, exhibiting a proportion of 74.5%, while the European region reported the lowest recorded proportion of 13.3% (Figure 4). Urinary tract infections reported the highest proportion of resistance, at 38.1%, while gastrointestinal tract reported the lowest proportion, at 60.9% (Supplementary File S2).

### Doxycycline

A total of 518 clinical isolates investigated in six studies were included in the analysis of doxycycline resistance. The estimated average proportion based on the random-effects model was 32.3% (95% CI, 15.2, 55.9) with substantial heterogeneity (I^2^ = 93.73%, *P* < 0.001) observed between included studies (Supplementary File S2; Figure 2).

### Chloramphenicol

A total of 3,076 clinical isolates investigated in 19 studies were included in the analysis of tetracycline resistance. The estimated average proportion based on the random-effects model was 16.9% (95% CI, 11.4, 24.4) with substantial heterogeneity (I^2^ = 93.53%, *P* < 0.001) observed between included studies (Supplementary File S2; Figure 2). The proportion of tetracycline resistance gradually decreased from 18.1% (95% CI 12.1–26.3) of 535 strains in 2000–2019 to 10% (95% CI 3.3–26.5) of 15 strains in 2020–2022 (Supplementary File S2; Figure 3; P = 0.365). Among 10 countries reporting resistance data for tetracycline, four (Kuwait, Italy, Hong Kong, and South Korea) reported that >25% of isolates had tetracycline resistance. There was no significant difference in the tetracycline resistance rates between countries (P = 0.940). The highest recorded proportion of resistance was observed in eastern Mediterranean western pacific region, exhibiting a proportion of 18.7% and 16.7%, while the African region reported the lowest recorded proportion of 2.6% (Figure 4). Urinary tract infections reported the highest proportion of resistance, at 40.3% (95% CI 12.4–76.2), while BSIs reported the lowest proportion, at 9.2% (95% CI 0.5–68.5) (Supplementary File S2).

### Erythromycin

A total of 4,784 clinical isolates investigated in 28 studies were included in the analysis of erythromycin resistance. The estimated average proportion based on the random-effects model was 62.8% (95% CI, 54.5, 70.4) with substantial heterogeneity (I^2^ = 95.84%, *P* < 0.001) observed between included studies (Supplementary File S2; Figure 2). The proportion of erythromycin resistance gradually increased from 66.6% in 2000–2019 to 55.4% in 2020–2022 (Supplementary File S2; Figure 2; P = 0.215). Among 17 countries reporting resistance data for erythromycin, four countries (Cuba, Italy, Germany, and Lebanon) reported that <45% of isolates had erythromycin resistance. There was an insignificant difference in the erythromycin resistance rates between WHO regional offices (P *=* 0.348). There was a significant difference in the resistance rates between AST methods (P *=* 0.036).

### Fosfomycin

A total of 495 clinical isolates investigated in five studies were included in the analysis of fosfomycin resistance. The estimated average proportion based on the random-effects model was 25.8% (95% CI, 5, 69.9) with significant heterogeneity (I^2^ = 93.95%, *P* < 0.001) observed between included studies (Supplementary File S2; Figure 2).

### Linezolid

A total of 5,040 clinical isolates investigated in 27 studies were included in the analysis of linezolid resistance. The estimated average proportion based on the random-effects model was 2% (95% CI, 0.5, 7.9) with substantial heterogeneity (I^2^ = 97.91%, *P* < 0.001) observed between included studies (Supplementary File S2; Figure 2). The proportion of linezolid resistance gradually decreased from 2.5% of 3,150 strains in 2000–2019 to 1.2% of 1,854 strains in 2020–2022 (Supplementary File S2; Figure 3; P *=* 0.556). Among 12 countries reporting resistance data for linezolid, two (Turkey and Indian) reported that >5% of isolates had linezolid resistance. There was no significant difference in the linezolid resistance rates between countries and WHO regional offices.

### Quinupristin-dalfopristin

A total of 1,397 clinical isolates investigated in 13 studies were included in the analysis of quinupristin-dalfopristin resistance. The estimated average proportion based on the random-effects model was 24.1% (95% CI, 9.1, 50.2) with substantial heterogeneity (I^2^ = 97.09%, *P* < 0.001) observed between included studies (Supplementary File S2; Figure 2). The proportion of quinupristin–dalfopristin resistance gradually increased from 23.1% in 2000–2019 to 28.2% in 2020–2022 (Supplementary File S2; Figure 3; P = 0.854). Six countries reported resistance data for quinupristin–dalfopristin. The subgroup analysis revealed a statistically significant disparity in the proportion of quinupristin–dalfopristin resistance among various countries, WHO regional offices, and AST methods (P *<* 0.029).

### Nitrofurantoin

A total of 2,598 clinical isolates investigated in 12 studies were included in the analysis of nitrofurantoin resistance. The estimated average proportion based on the random-effects model was 38.5% (95% CI, 29.1, 49) with substantial heterogeneity (I^2^ = 94.5%, *P* < 0.001) observed between included studies (Supplementary File S2; Figure 2). The proportion of nitrofurantoin resistance gradually increased from 35.4% in 2000–2019 to 41.9% in 2020–2022 (Supplementary File S2; Figure 3; P = 0.618). There was a significant difference in the nitrofurantoin resistance rates between countries and WHO regional offices (P < 0.001).

### Rifampicin

A total of 2,133 clinical isolates investigated in 11 studies were included in the analysis of rifampicin resistance. The estimated average proportion based on the random-effects model was 49.1% (95% CI, 31.5, 67) with substantial heterogeneity (I^2^ = 97.34%, *P* < 0.001) observed between included studies (Supplementary File S2; Figure 2). The proportion of rifampicin resistance significantly increased from 38.7% in 2000–2019 to 76.1% in 2020–2022 (Supplementary File S2; Figure 3; P *=* 036). There was a significant difference in the rifampicin resistance rates over time and between WHO regional offices (P < 0.036).

### Trimthoprim–sulfamethoxazole

A total of 666 clinical isolates investigated in eight studies were included in the analysis of rifampicin resistance. The estimated average proportion based on the random-effects model was 61.8% (95% CI, 40.6, 79.3) with substantial heterogeneity (I^2^ = 92.81%, *P* < 0.001) observed between included studies (Supplementary File S2; Figure 2).

## Discussion

Understanding the prevalence of antibiotic resistance is essential for developing effective strategies to prevent its spread. Globally, the number of infections caused by *E. faecium* has increased significantly in recent years (Melese et al., 2020). Previous studies and systematic reviews on enterococci investigated prevalence and resistance; however, all of the research focused on localized prevalence or resistance, specifically examining vancomycin resistance, or included *enterococci* strains isolated from specific infections, such as BSIs or UTIs (Emaneini et al., 2016; Kilbas and Ciftci, 2018; Correa-Martínez et al., 2021). However, a comprehensive review of *E. faecium* resistance to different antibiotics across various infections has not yet been conducted. This study aimed to determine the global antimicrobial resistance profile of enterococci in different infections. This systematic review and meta-analysis included 56 eligible studies on antibiotic resistance in *E. faecium*, published between 2000 and 2022. Glycopeptides, including vancomycin and teicoplanin, are among the last-resort options in our arsenal against Gram-positive bacteria (Jakaria et al., 2022). However, resistance to this class has been reported in *E. faecium* isolates (Melese et al., 2020)*.* According to our results, the overall resistance to vancomycin and teicoplanin in *E. faecium* was 14%. European studies and the National Antimicrobial Resistance Surveillance of Turkey (NAMRS-T) reported resistance rates of approximately 11% and 17%, respectively (Agus et al., 2006; Kilbas and Ciftci, 2018). Infection control programs and adherence to hand hygiene by healthcare workers are likely the most effective strategies for reducing the prevalence and resistance of *E. faecium* (Kilbas and Ciftci, 2018).

Beta-lactams remain widely prescribed against infections due to their broad spectrum of activity, established efficacy, and safety profile. Despite high resistance rates in certain pathogens like *E. faecium*, they are often used as first-line treatments, particularly when susceptibility is confirmed or in combination therapy to enhance efficacy. Among them, penicillin, ampicillin, and imipenem show the greatest potency, but cephalosporins, as monotherapy, have no activity against *E. faecium* (Miller et al., 2020). However, susceptibility to these effective antibiotics has decreased in recent decades. Results of the present review demonstrated that the resistance rate to nearly all investigated members of the beta-lactam group (penicillin, ampicillin, and imipenem) was more than 57%. This high resistance rate is in accordance with meta-analyses performed by Kilbas et al. in Turkey, where their data were collected from 2000 to 2015 (Kilbas and Ciftci, 2018). This high resistance shows the necessity to create new guidelines for treating *E. faecium* infections and replace these antibiotics with others. However, resistance against amoxicillin-clavulanic acid was lower than to other antibiotics in the beta-lactam group (24%). It demonstrates that beta-lactamase enzymes play an important role in *E. faecium* strains that are resistant to the beta-lactam class, and implementing beta-lactamase inhibitors can be used as an option for successful treatment and prevention of increased resistance.

Aminoglycosides encompass a wide range of antibiotics. Among them, gentamicin stands out as the most effective against *E. faecium* when used synergistically with β-lactams (Miller et al., 2020). However, the mechanism of reduced drug uptake by bacterial cells leads to a low-level susceptibility within this class of antibiotics (Kilbas and Ciftci, 2018). Our analysis demonstrates that approximately half of the investigated strains showed resistance to gentamycin and/or streptomycin. The presence of aminoglycoside modifying enzymes (AMEs), such as AAC (6′)-Ie-APH(2″)Ia enzyme and adenyltransferase, confers resistance to these agents (Torres et al., 2018; Jubeh et al., 2020; Miller et al., 2020).

The tetracycline family includes several active agents used against both Gram-negative and Gram-positive bacteria (Torres et al., 2018). In accordance with a meta-analysis reported by Melese et al. (2020) in Ethiopia, a high rate of resistance to tetracycline and doxycycline was observed in the present study. Often, the use of tetracycline and doxycycline in veterinary and human medicine has led to the development of resistance to these antibiotics (Garcia-Migura et al., 2014). The best-known mechanisms of tetracycline resistance in *E. faecium* are ribosomal protection mediated by genes such as *tet(M)*, *tet(O)*, and *tet(S)*. These mechanisms have been extensively documented (Chopra I and Roberts, 2001; Fiedler et al., 2016). However, in the current study, resistance to tigecycline, a new generation of tetracyclines, is very low (0.5%). Although most efflux pumps and ribosomal protection proteins do not affect tigecycline, a previous study suggests the role of tet(L) and tet(M) in the resistance of *E. faecium* (Fiedler et al., 2016; Miller et al., 2020).

Ciprofloxacin, levofloxacin, and moxifloxacin agents are the most used compounds in the fluoroquinolone family (Torres et al., 2018). This family inhibits bacterial protein synthesis by affecting DNA gyrase. Generally, mutations in gyrase enzymes and efflux pumps are associated with resistance to this class of antibiotics (Mirzaii et al., 2023). The elevated resistance rates among fluoroquinolones align with findings from meta-analyses conducted in Turkey (Kilbas and Ciftci, 2018) and China (Yan et al., 2023). Most likely, misuse of these antimicrobial agents and pressure selection have a vital role in the development and distribution of resistant strains (Alcalde-Rico et al., 2016). However, in another meta-analysis (Miller et al., 2020), the frequency of resistance was lower than that reported by the current study. The most likely reason for the variation in the prevalence of resistant strains is related to differences in antibiotic-prescribed patterns (Mirzaii et al., 2023).

The newer agent linezolid is the only antibiotic integrated into the oxazolidinone family that has been approved by the FDA, and it is capable of preventing bacterial protein synthesis. Generally, the frequency of resistance is very low (Kim and Koo, 2020; Mirzaii et al., 2023); thus, this agent is prescribed against infection caused by enterococci strains (Miller et al., 2020). After analyzing 5,040 isolates in 27 studies in this review, a low resistance rate was illustrated that was relatively similar to those reported by Kilbas and Ciftci (2018). However, a higher rate was reported from Ethiopia, but 30 isolates and two studies were included in that study (Melese et al., 2020).

In addition, based on our analysis, the resistance rate to vancomycin and teicoplanin varied in different regions, such that the western Pacific region and European regions showed the highest and the lowest proportion of resistance to vancomycin, respectively. The highest recorded proportion of resistance was observed in the western Pacific region. This region’s unique resistance drivers, including high population density, widespread antibiotic use in agriculture and healthcare, and variable access to antimicrobial stewardship programs, likely contribute to these elevated rates.

In addition, subgroup analysis revealed a gradual increase in resistance rates in some agents like glycopeptides, ampicillin, streptomycin, levofloxacin, and erythromycin. Overuse and history of antimicrobial exposure are major reasons for increasing and local differences in resistance rates (Emaneini et al., 2016). Conversely, a decrease in resistance rates was observed for penicillin, gentamycin, and chloramphenicol.

Although antibiotic resistance poses a significant public health threat, certain conditions can enable bacteria to regain susceptibility to antibiotics. Reducing antibiotic usage and optimizing prescribing practices can lessen selection pressure, promoting the resurgence and spread of susceptible bacterial strains. In agreement with another study (Melese et al., 2020), our findings also demonstrated that the highest resistance to most antimicrobial agents was observed in isolates recovered from UTIs. Notably, over one-third of the *E. faecium* isolates from UTIs were resistant to vancomycin. Given that the majority of urinary infections caused by *E. faecium* are nosocomial in nature, the extensive use of vancomycin in hospitals can lead to increased selection pressure on nosocomial pathogens, resulting in greater resistance to this antibiotic (Yan et al., 2023). Among the 20 countries reporting vancomycin resistance data, 13 nations observed that more than 5% of their isolates presented resistance to this critical antibiotic. Variations in healthcare systems, infection control measures, and antibiotic stewardship initiatives across these nations may contribute to the diverse resistance rates. Countries with strong infection control practices may report lower resistance rates, while those with higher antibiotic consumption or inadequate surveillance systems might experience a more significant prevalence.

In addition to acquired resistance mechanisms, *E*. *faecium* is naturally resistant to several classes of antibiotics, including cephalosporins, low levels of aminoglycosides, and lincosamides. These intrinsic resistance mechanisms result from structural features of the bacterium, such as the lack of high-affinity penicillin-binding proteins and alterations in ribosomal target sites. This natural resistance significantly limits the range of antibiotics that can be used to treat *E. faecium* infections and complicates treatment regimens, particularly when *E. faecium* is co-resistant to multiple other agents.

The inherent resistance of *E. faecium* to beta-lactams, aminoglycosides, and lincosamides must be considered when reviewing secondary data on antibiotic resistance patterns. While the focus of this study was on acquired resistance, it is important to note that the baseline, intrinsic resistance of *E. faecium* plays a crucial role in shaping the overall resistance landscape. This natural resistance should not be overlooked, as it influences the effectiveness of antibiotic therapies, especially in regions where the prevalence of *E. faecium* infections is high. The failure to account for intrinsic resistance mechanisms during the review of secondary data may lead to misinterpretations of the true scope of acquired resistance in *E. faecium*. Therefore, it is essential that future studies incorporate both acquired and intrinsic resistance data to provide a more comprehensive understanding of the challenges posed by *E. faecium* and to help guide the development of more effective treatment strategies.

Understanding intrinsic resistance is critical for designing effective antimicrobial stewardship programs. By recognizing the baseline resistance of *E. faecium*, clinicians can make more informed decisions regarding empirical treatment choices, particularly in high-risk patients, such as those in intensive care units. Additionally, the identification of intrinsic resistance highlights the importance of early detection and targeted antimicrobial therapy, which can help avoid unnecessary use of broad-spectrum antibiotics and minimize the development of acquired resistance.

Given the challenges posed by *E. faecium*, there is an urgent need for novel therapeutic strategies that target its intrinsic resistance mechanisms. Further research into the structural features that confer this natural resistance could lead to the development of new treatments that can overcome these barriers, potentially restoring the efficacy of previously ineffective antibiotics.

Our findings confirm the high efficacy of linezolid against *E. faecium*, emphasizing its role as a key treatment for MDR infections, including BSIs and endocarditis. This underscores the importance of antimicrobial stewardship programs to preserve its effectiveness and informs evidence-based treatment protocols. Additionally, the data highlight the need for ongoing surveillance to guide empirical therapy and public health policies targeting *E. faecium*-related infections. A key strength of this study lies in its comprehensive search strategy and clearly defined inclusion and exclusion criteria.

While our analysis focused on resistance patterns to individual antibiotics, it is essential to also consider the multidrug resistance (MDR) profiles of *E. faecium* isolates. Resistance to multiple classes of antibiotics in *E. faecium* complicates treatment choices, especially when isolates are resistant to commonly used first-line agents. Our study will now include an analysis of the co-occurrence of resistance to multiple antibiotics, highlighting the prevalence of MDR strains. For example, *E. faecium* isolates resistant to vancomycin were frequently also resistant to other antibiotics, such as aminoglycosides and macrolides, leading to significant challenges in selecting effective therapies. This pattern of multidrug resistance, observed in both hospital and community-acquired infections, underscores the importance of screening for MDR in clinical settings.

The identification of MDR strains is critical for selecting appropriate treatment regimens. Given the rise of multidrug resistance, physicians should consider combination therapies that target multiple resistance mechanisms, particularly in severe infections where monotherapy may not be effective. For instance, using linezolid or daptomycin in combination with other antibiotics may provide better clinical outcomes for patients infected with MDR *E. faecium*. Future studies should investigate the interactions between antibiotics in multidrug-resistant infections to guide evidence-based recommendations for combination therapies. Monitoring and incorporating MDR profiles into routine clinical practice will help ensure more effective treatment options and prevent further escalation of resistance.

The primary limitation of this study is the potential bias in resistance rates due to the combination of resistance data from patients of different genders. Additionally, our analysis is constrained by the lack of detailed proportion data distinguishing between *E. faecium* colonization and infection. Colonization frequently precedes infection, particularly in hospital settings where antibiotic pressure and compromised immune systems are significant contributing factors. To better understand the clinical impact of *E. faecium*, future research should prioritize clarifying colonization rates and their progression to infection. In this study, both CLSI and EUCAST standards were reported in the included articles, reflecting the diversity in laboratory practices globally. Resistance data were presented as per the original studies without direct comparison between the two standards to avoid inconsistencies arising from differences in breakpoints. While this approach captures the real-world variation, it underscores the need for globally harmonized antimicrobial susceptibility testing criteria to ensure comparability and consistency in reporting.

Another limitation of this meta-analysis is the lack of stratification based on the source of *E. faecium* isolates (e.g., ICU, outpatient, and general hospital wards) in the studies included. The resistance patterns of *E. faecium* may vary considerably depending on the clinical setting of the patient, and this factor was not sufficiently accounted for. Future studies should aim to include these variables to provide more accurate data on antibiotic resistance patterns and to refine strategies for antimicrobial stewardship in different healthcare settings.

This study primarily analyzed phenotypic resistance patterns, but it is important to recognize the genetic mechanisms behind *E. faecium* resistance. Key resistance genes, such as *vanA* (vancomycin), *ermB* (macrolides), and aac (6′)-Ie-aph (2″) (aminoglycosides), contribute significantly to acquired resistance. However, many studies did not report molecular data, which limits our understanding of the genetic underpinnings of resistance. Incorporating molecular techniques like PCR and genome sequencing in future studies will provide a clearer picture of the genetic basis of *E. faecium* resistance, allowing for better-targeted antimicrobial therapies.

## Conclusion

Our findings confirm the high antibacterial activity of linezolid against *E. faecium* isolates. This highlights the importance of preserving linezolid as a key therapeutic option through antimicrobial stewardship programs, ensuring its prioritization for treating MDR *E. faecium* infections, particularly in settings where alternative treatment options are limited. Moreover, our investigation reveals a gradual increase and a troubling upward trend in the resistance rates of almost all agents in recent years. These results highlight the importance of continuous surveillance, a practice that should be applied by policymakers. However, our analysis showed a significant reduction in resistance rates for some antibiotics, so these drugs can make a strong comeback in the future.

## Data Availability

The original contributions presented in the study are included in the article/Supplementary Material; further inquiries can be directed to the corresponding authors.
