# Supplementary material for: Global trends in antimicrobial resistance of Enterococcus faecium: a systematic review and meta-analysis of clinical isolates
Source: Front Pharmacol. 2025 Apr 7;16:1505674. doi: 10.3389/fphar.2025.1505674 (PMC12009923; doi:10.3389/fphar.2025.1505674)

**Supplementary Table S1.** The detailed meta-analysis data in this study.

| **Antibiotic** | **Category** | **Subgroup** | **K (n. N)** | **Proportion (LCI, HCI)** | **I^2^** | **P. value** |
| --- | --- | --- | --- | --- | --- | --- |
| **vancomycin** | Overall | NA | 46 (1597, 5560) | 0.140 (0.085, 0.222) | 96.45% | NA |
|  | year group | 2000_2019 | 29 (1235, 3395) | 0.133 (0.064, 0.254) | 96.84% | 0.741 |
|  |  | 2020_2024 | 16 (362, 2129) | 0.175 (0.094, 0.303) | 92.93% |  |
|  | country | Taiwan | 2 (75, 75) | 0.986 (0.909, 0.998) | 0.00% | <0.001 |
|  |  | Lebanon | 1 (0, 35) | 0.014 (0.001, 0.187) | 0.00% |  |
|  |  | Kuwait | 1 (7, 354) | 0.020 (0.009, 0.041) | 0.00% |  |
|  |  | Germany | 1 (1, 45) | 0.022 (0.003, 0.142) | 0.00% |  |
|  |  | India | 7 (39, 271) | 0.115 (0.041, 0.284) | 80.81% |  |
|  |  | Turkey | 3 (4, 114) | 0.041 (0.016, 0.099) | 0.00% |  |
|  |  | Cuba | 1 (1, 10) | 0.100 (0.014, 0.467) | 0.00% |  |
|  |  | South Korea | 2 (57, 493) | 0.086 (0.004, 0.684) | 98.07% |  |
|  |  | Italy | 3 (16, 878) | 0.012 (0.002, 0.060) | 55.54% |  |
|  |  | Iran | 7 (179, 524) | 0.316 (0.164, 0.521) | 93.06% |  |
|  |  | US | 3 (12, 249) | 0.136 (0.006, 0.800) | 89.02% |  |
|  |  | Brazil | 1 (0, 4) | 0.100 (0.006, 0.674) | 0.00% |  |
|  |  | Nigeria | 1 (1, 1) | 0.750 (0.109, 0.987) | 0.00% |  |
|  |  | Poland | 3 (53, 146) | 0.191 (0.037, 0.593) | 70.19% |  |
|  |  | Algeria | 1 (0, 39) | 0.013 (0.001, 0.171) | 0.00% |  |
|  |  | China | 5 (912, 1691) | 0.072 (0.008, 0.412) | 96.52% |  |
|  |  | Hungary | 1 (71, 71) | 0.993 (0.899, 1.000) | 0.00% |  |
|  |  | Romania | 1 (0, 26) | 0.019 (0.001, 0.236) | 0.00% |  |
|  |  | Israel | 1 (14, 46) | 0.304 (0.189, 0.451) | 0.00% |  |
|  |  | Australia | 1 (155, 488) | 0.318 (0.278, 0.360) | 0.00% |  |
|  | who.regional.offices | western pacific region | 9 (1044, 2259) | 0.231 (0.057, 0.599) | 98.13% | 0.725 |
|  |  | eastern mediterranean region | 9 (186, 913) | 0.202 (0.084, 0.411) | 95.37% |  |
|  |  | european region | 14 (314, 1814) | 0.093 (0.041, 0.195) | 93.21% |  |
|  |  | south-east asia region | 7 (39, 271) | 0.115 (0.041, 0.284) | 80.81% |  |
|  |  | region of the americas | 5 (13, 263) | 0.128 (0.022, 0.494) | 78.69% |  |
|  |  | african region | 2 (1, 40) | 0.155 (0.001, 0.975) | 84.31% |  |
|  | ast_classification | CLSI | 40 (1369, 4925) | 0.135 (0.075, 0.231) | 96.71% | 0.301 |
|  |  | Multiple Standards | 2 (156, 510) | 0.158 (0.021, 0.625) | 79.66% |  |
|  |  | EUCAST | 2 (72, 89) | 0.732 (0.001, 1.000) | 94.94% |  |
|  | infection.source | Bloodstream | 10 (257, 1109) | 0.188 (0.070, 0.414) | 91.48% | 0.189 |
|  |  | Mixed | 25 (1027, 3325) | 0.071 (0.026, 0.180) | 97.46% |  |
|  |  | urinary tract | 6 (224, 560) | 0.374 (0.228, 0.548) | 91.43% |  |
|  |  | Gastrointestinal tract | 3 (71, 525) | 0.207 (0.000, 0.996) | 94.28% |  |
|  | ast.methods | MIC based methods | 18 (371, 2325) | 0.132 (0.066, 0.246) | 90.94% | 0.689 |
|  |  | Disc Diffusion | 28 (1226, 3235) | 0.148 (0.074, 0.275) | 97.17% |  |
| **gentamicin** | Overall | NA | 31 (2333, 5077) | 0.504 (0.418, 0.589) | 95.86% | NA |
|  | year_group | 2000_2019 | 21 (1683, 3618) | 0.537 (0.430, 0.640) | 96.25% | 0.262 |
|  |  | 2020_2024 | 10 (650, 1459) | 0.421 (0.268, 0.592) | 95.29% |  |
|  | country | Taiwan | 1 (25, 50) | 0.500 (0.365, 0.635) | 0.00% | 0.234 |
|  |  | Lebanon | 1 (2, 35) | 0.057 (0.014, 0.202) | 0.00% |  |
|  |  | Kuwait | 1 (52, 354) | 0.147 (0.114, 0.188) | 0.00% |  |
|  |  | India | 3 (59, 76) | 0.768 (0.583, 0.887) | 45.20% |  |
|  |  | Hong Kong | 1 (13, 35) | 0.371 (0.229, 0.540) | 0.00% |  |
|  |  | Turkey | 3 (90, 163) | 0.636 (0.136, 0.951) | 96.56% |  |
|  |  | Cuba | 1 (3, 10) | 0.300 (0.100, 0.624) | 0.00% |  |
|  |  | South Korea | 1 (178, 330) | 0.539 (0.485, 0.593) | 0.00% |  |
|  |  | Italy | 2 (449, 810) | 0.514 (0.295, 0.729) | 97.35% |  |
|  |  | US | 2 (150, 672) | 0.358 (0.104, 0.727) | 94.66% |  |
|  |  | Iran | 6 (324, 477) | 0.679 (0.481, 0.828) | 92.77% |  |
|  |  | Nigeria | 1 (1, 1) | 0.750 (0.109, 0.987) | 0.00% |  |
|  |  | Poland | 3 (72, 146) | 0.435 (0.111, 0.825) | 79.82% |  |
|  |  | Algeria | 1 (24, 39) | 0.615 (0.456, 0.753) | 0.00% |  |
|  |  | China | 2 (618, 1139) | 0.095 (0.000, 0.962) | 93.60% |  |
|  |  | Australia | 1 (140, 488) | 0.287 (0.248, 0.329) | 0.00% |  |
|  | who.regional.offices | western pacific region | 5 (834, 1554) | 0.493 (0.393, 0.593) | 83.81% | 0.298 |
|  |  | eastern Mediterranean region | 8 (378, 866) | 0.508 (0.256, 0.756) | 97.29% |  |
|  |  | south-east Asia region | 3 (59, 76) | 0.768 (0.583, 0.887) | 45.20% |  |
|  |  | European region | 10 (884, 1859) | 0.495 (0.365, 0.626) | 95.44% |  |
|  |  | region of the Americas | 3 (153, 682) | 0.340 (0.137, 0.628) | 89.49% |  |
|  |  | African region | 2 (25, 40) | 0.621 (0.466, 0.755) | 0.00% |  |
|  | ast_classification | CLSI | 28 (2167, 4532) | 0.523 (0.432, 0.612) | 95.77% | 0.184 |
|  |  | EUCAST | 1 (2, 18) | 0.111 (0.028, 0.352) | 0.00% |  |
|  |  | Multiple Standards | 1 (140, 488) | 0.287 (0.248, 0.329) | 0.00% |  |
|  | infection.source | Bloodstream | 6 (386, 936) | 0.519 (0.363, 0.671) | 92.55% | 0.208 |
|  |  | Mixed | 19 (1670, 3730) | 0.447 (0.335, 0.565) | 96.79% |  |
|  |  | Gastrointestinal tract | 1 (13, 35) | 0.371 (0.229, 0.540) | 0.00% |  |
|  |  | urinary tract | 4 (263, 375) | 0.707 (0.535, 0.835) | 88.17% |  |
|  | ast.methods | MIC based methods | 12 (853, 2240) | 0.502 (0.392, 0.612) | 95.07% | 0.985 |
|  |  | Disc Diffusion | 19 (1480, 2837) | 0.500 (0.380, 0.620) | 95.24% |  |
| **Amoxicillin clavulanic acid** | Overall | NA | 4 (42, 195) | 0.204 (0.091, 0.397) | 83.14% | NA |
| **ampicillin** | Overall | NA | 40 (3110, 5720) | 0.589 (0.465, 0.703) | 97.43% | NA |
|  | year_group | 2000_2019 | 28 (1506, 3585) | 0.512 (0.382, 0.640) | 96.42% | 0.083 |
|  |  | 2020_2024 | 12 (1604, 2135) | 0.723 (0.509, 0.868) | 97.14% |  |
|  | country | Lebanon | 1 (5, 35) | 0.143 (0.061, 0.300) | 0.00% | 0.030 |
|  |  | Kuwait | 1 (29, 354) | 0.082 (0.058, 0.115) | 0.00% |  |
|  |  | India | 4 (111, 176) | 0.655 (0.375, 0.857) | 88.09% |  |
|  |  | Hong Kong | 1 (11, 35) | 0.314 (0.183, 0.483) | 0.00% |  |
|  |  | Turkey | 3 (112, 118) | 0.950 (0.659, 0.995) | 78.11% |  |
|  |  | Cuba | 1 (5, 10) | 0.500 (0.225, 0.775) | 0.00% |  |
|  |  | South Korea | 2 (65, 493) | 0.130 (0.018, 0.547) | 97.91% |  |
|  |  | Italy | 3 (482, 878) | 0.236 (0.010, 0.908) | 99.25% |  |
|  |  | Iran | 7 (301, 749) | 0.434 (0.183, 0.725) | 96.93% |  |
|  |  | US | 3 (168, 249) | 0.625 (0.300, 0.866) | 90.21% |  |
|  |  | Brazil | 1 (0, 4) | 0.100 (0.006, 0.674) | 0.00% |  |
|  |  | Nigeria | 1 (1, 1) | 0.750 (0.109, 0.987) | 0.00% |  |
|  |  | China | 6 (1254, 1772) | 0.912 (0.740, 0.974) | 97.47% |  |
|  |  | Poland | 2 (6, 21) | 0.476 (0.028, 0.966) | 78.75% |  |
|  |  | Algeria | 1 (37, 39) | 0.949 (0.817, 0.987) | 0.00% |  |
|  |  | Israel | 1 (40, 46) | 0.870 (0.739, 0.940) | 0.00% |  |
|  |  | Australia | 1 (428, 488) | 0.877 (0.845, 0.903) | 0.00% |  |
|  | who.regional.offices | eastern Mediterranean region | 9 (335, 1138) | 0.337 (0.128, 0.636) | 97.76% | 0.277 |
|  |  | south-east Asia region | 4 (111, 176) | 0.655 (0.375, 0.857) | 88.09% |  |
|  |  | western pacific region | 9 (1330, 2300) | 0.721 (0.466, 0.884) | 98.17% |  |
|  |  | European region | 11 (1123, 1803) | 0.646 (0.352, 0.860) | 98.22% |  |
|  |  | region of the Americas | 5 (173, 263) | 0.535 (0.281, 0.772) | 84.25% |  |
|  |  | african region | 2 (38, 40) | 0.931 (0.775, 0.981) | 3.49% |  |
|  | ast_classification | CLSI | 36 (2625, 5153) | 0.567 (0.437, 0.688) | 97.38% | 0.144 |
|  |  | Multiple Standards | 2 (445, 510) | 0.853 (0.751, 0.918) | 49.40% |  |
|  |  | EUCAST | 1 (3, 18) | 0.167 (0.055, 0.409) | 0.00% |  |
|  | infection.source | Mixed | 25 (1881, 3591) | 0.598 (0.449, 0.731) | 97.09% | 0.589 |
|  |  | Gastrointestinal tract | 4 (430, 724) | 0.278 (0.016, 0.901) | 98.70% |  |
|  |  | Bloodstream | 6 (524, 940) | 0.607 (0.177, 0.917) | 98.41% |  |
|  |  | urinary tract | 4 (236, 425) | 0.615 (0.331, 0.838) | 96.21% |  |
|  | ast.methods | Disc Diffusion | 24 (1694, 3207) | 0.535 (0.388, 0.677) | 96.68% | 0.333 |
|  |  | MIC based methods | 16 (1416, 2513) | 0.679 (0.445, 0.848) | 98.18% |  |
| **chloramphenicol** | Overall | NA | 19 (550, 3076) | 0.169 (0.114, 0.244) | 93.53% | NA |
|  | year_group | 2000_2019 | 16 (535, 2830) | 0.181 (0.121, 0.263) | 93.95% | 0.365 |
|  |  | 2020_2024 | 3 (15, 246) | 0.100 (0.033, 0.265) | 48.51% |  |
|  | country | Lebanon | 1 (4, 35) | 0.114 (0.044, 0.268) | 0.00% | 0.940 |
|  |  | Kuwait | 1 (106, 354) | 0.299 (0.254, 0.349) | 0.00% |  |
|  |  | Hong Kong | 1 (12, 35) | 0.343 (0.206, 0.512) | 0.00% |  |
|  |  | India | 3 (18, 85) | 0.152 (0.019, 0.618) | 67.56% |  |
|  |  | South Korea | 1 (88, 330) | 0.267 (0.222, 0.317) | 0.00% |  |
|  |  | Italy | 1 (84, 260) | 0.323 (0.269, 0.382) | 0.00% |  |
|  |  | Brazil | 1 (0, 4) | 0.100 (0.006, 0.674) | 0.00% |  |
|  |  | Iran | 6 (105, 575) | 0.176 (0.058, 0.428) | 95.41% |  |
|  |  | Algeria | 1 (1, 39) | 0.026 (0.004, 0.161) | 0.00% |  |
|  |  | China | 2 (116, 1107) | 0.087 (0.043, 0.170) | 52.30% |  |
|  | who.regional.offices | eastern mediterranean region | 8 (215, 964) | 0.187 (0.094, 0.339) | 93.86% | 0.800 |
|  |  | western pacific region | 4 (216, 1472) | 0.167 (0.081, 0.315) | 95.04% |  |
|  |  | south-east asia region | 3 (18, 85) | 0.152 (0.019, 0.618) | 67.56% |  |
|  |  | european region | 2 (100, 512) | 0.154 (0.026, 0.552) | 97.79% |  |
|  |  | region of the americas | 1 (0, 4) | 0.100 (0.006, 0.674) | 0.00% |  |
|  |  | african region | 1 (1, 39) | 0.026 (0.004, 0.161) | 0.00% |  |
|  | ast_classification | CLSI | 18 (549, 3037) | 0.179 (0.121, 0.257) | 93.77% | 0.119 |
|  | Infection source | Mixed | 11 (384, 2337) | 0.150 (0.091, 0.237) | 94.41% | 0.364 |
|  |  | Gastrointestinal tract | 3 (25, 274) | 0.137 (0.025, 0.493) | 91.30% |  |
|  |  | Bloodstream | 2 (88, 363) | 0.092 (0.005, 0.685) | 79.94% |  |
|  |  | urinary tract | 2 (53, 101) | 0.403 (0.124, 0.762) | 74.77% |  |
|  | AST methods | Disc Diffusion | 12 (347, 2117) | 0.170 (0.096, 0.283) | 94.77% | 0.843 |
|  |  | MIC based methods | 7 (203, 959) | 0.169 (0.094, 0.285) | 89.75% |  |
| **ciprofloxacin** | Overall | NA | 34 (1785, 3295) | 0.630 (0.542, 0.710) | 94.31% | NA |
|  | year_group | 2000_2019 | 24 (1224, 2190) | 0.635 (0.522, 0.735) | 94.59% | 0.763 |
|  |  | 2020_2024 | 9 (534, 1069) | 0.600 (0.423, 0.754) | 94.57% |  |
|  | country | Taiwan | 1 (18, 50) | 0.360 (0.240, 0.501) | 0.00% | 0.409 |
|  |  | Lebanon | 1 (12, 35) | 0.343 (0.206, 0.512) | 0.00% |  |
|  |  | Kuwait | 1 (140, 354) | 0.395 (0.346, 0.447) | 0.00% |  |
|  |  | India | 6 (172, 220) | 0.774 (0.706, 0.830) | 14.17% |  |
|  |  | Hong Kong | 1 (20, 35) | 0.571 (0.406, 0.723) | 0.00% |  |
|  |  | Cuba | 1 (3, 10) | 0.300 (0.100, 0.624) | 0.00% |  |
|  |  | South Korea | 2 (210, 493) | 0.501 (0.133, 0.867) | 98.75% |  |
|  |  | Italy | 2 (99, 328) | 0.301 (0.249, 0.358) | 8.06% |  |
|  |  | US | 2 (35, 38) | 0.910 (0.771, 0.968) | 0.00% |  |
|  |  | Iran | 7 (360, 668) | 0.696 (0.430, 0.874) | 96.67% |  |
|  |  | Brazil | 1 (2, 4) | 0.500 (0.123, 0.877) | 0.00% |  |
|  |  | Nigeria | 1 (1, 1) | 0.750 (0.109, 0.987) | 0.00% |  |
|  |  | Poland | 1 (0, 3) | 0.125 (0.007, 0.734) | 0.00% |  |
|  |  | Turkey | 3 (115, 160) | 0.735 (0.475, 0.895) | 87.46% |  |
|  |  | China | 2 (83, 156) | 0.532 (0.453, 0.609) | 0.00% |  |
|  |  | Australia | 1 (281, 488) | 0.576 (0.531, 0.619) | 0.00% |  |
|  | who.regional.offices | western pacific region | 6 (331, 734) | 0.501 (0.330, 0.672) | 94.46% | 0.510 |
|  |  | eastern Mediterranean region | 9 (512, 1057) | 0.615 (0.427, 0.774) | 95.85% |  |
|  |  | south-east Asia region | 6 (172, 220) | 0.774 (0.706, 0.830) | 14.17% |  |
|  |  | region of the Americas | 4 (40, 52) | 0.709 (0.287, 0.937) | 77.94% |  |
|  |  | European region | 8 (729, 1231) | 0.608 (0.406, 0.780) | 96.46% |  |
|  |  | African region | 1 (1, 1) | 0.750 (0.109, 0.987) | 0.00% |  |
|  | ast_classification | CLSI | 31 (1461, 2749) | 0.626 (0.524, 0.717) | 94.62% | 0.901 |
|  |  | Multiple Standards | 2 (297, 510) | 0.618 (0.475, 0.742) | 47.89% |  |
|  | infection.source | Bloodstream | 8 (518, 1030) | 0.611 (0.435, 0.763) | 94.36% | 0.168 |
|  |  | Mixed | 18 (926, 1646) | 0.630 (0.506, 0.739) | 94.12% |  |
|  |  | Gastrointestinal tract | 3 (70, 274) | 0.391 (0.142, 0.714) | 90.05% |  |
|  |  | urinary tract | 4 (270, 344) | 0.789 (0.682, 0.867) | 64.85% |  |
|  | ast.methods | MIC based methods | 15 (1065, 1846) | 0.704 (0.571, 0.810) | 95.75% | 0.119 |
|  |  | Disc Diffusion | 19 (720, 1449) | 0.564 (0.444, 0.677) | 92.54% |  |
| **doxycycline** | Overall | NA | 6 (132, 518) | 0.323 (0.152, 0.559) | 93.73% | NA |
| **fosfomycin** | Overall | NA | 4 (29, 471) | 0.121 (0.024, 0.438) | 92.63% | NA |
| **imipenem** | Overall | NA | 9 (766, 1076) | 0.570 (0.357, 0.759) | 96.08% | NA |
| **levofloxacin** | Overall | NA | 13 (2214, 2965) | 0.751 (0.640, 0.837) | 94.40% | NA |
|  | year_group | 2000_2019 | 10 (1686, 2381) | 0.630 (0.535, 0.717) | 86.33% | <0.001 |
|  |  | 2020_2024 | 3 (528, 584) | 0.927 (0.852, 0.966) | 59.25% |  |
|  | country | Germany | 1 (24, 45) | 0.533 (0.389, 0.672) | 0.00% | 0.087 |
|  |  | Hong Kong | 1 (18, 35) | 0.514 (0.353, 0.673) | 0.00% |  |
|  |  | Cuba | 1 (0, 10) | 0.045 (0.003, 0.448) | 0.00% |  |
|  |  | US | 2 (776, 843) | 0.978 (0.074, 1.000) | 95.13% |  |
|  |  | Algeria | 1 (38, 39) | 0.974 (0.839, 0.996) | 0.00% |  |
|  |  | China | 5 (1149, 1691) | 0.824 (0.602, 0.936) | 97.61% |  |
|  |  | India | 1 (32, 50) | 0.640 (0.499, 0.760) | 0.00% |  |
|  | who.regional.offices | European region | 2 (201, 297) | 0.633 (0.460, 0.777) | 79.41% | 0.473 |
|  |  | western pacific region | 6 (1167, 1726) | 0.781 (0.573, 0.905) | 97.05% |  |
|  |  | region of the Americas | 3 (776, 853) | 0.828 (0.061, 0.997) | 92.71% |  |
|  |  | African region | 1 (38, 39) | 0.974 (0.839, 0.996) | 0.00% |  |
|  |  | south-east Asia region | 1 (32, 50) | 0.640 (0.499, 0.760) | 0.00% |  |
|  | ast_classification | CLSI | 12 (2176, 2926) | 0.729 (0.613, 0.821) | 94.64% | 0.047 |
|  | infection.source | Mixed | 10 (1757, 2440) | 0.703 (0.596, 0.792) | 90.38% | 0.666 |
|  |  | Gastrointestinal tract | 2 (418, 485) | 0.749 (0.291, 0.956) | 96.65% |  |
|  | ast.methods | Disc Diffusion | 6 (766, 1306) | 0.618 (0.460, 0.754) | 88.08% | 0.018 |
|  |  | MIC based methods | 7 (1448, 1659) | 0.856 (0.730, 0.929) | 93.49% |  |
| **linezolid** | Overall | NA | 27 (993, 5040) | 0.020 (0.005, 0.079) | 97.91% | NA |
|  | year_group | 2000_2019 | 17 (974, 3150) | 0.025 (0.004, 0.139) | 98.24% | 0.556 |
|  |  | 2020_2024 | 9 (14, 1854) | 0.012 (0.005, 0.031) | 62.16% |  |
|  | country | Taiwan | 2 (0, 75) | 0.014 (0.002, 0.091) | 0.00% | 0.982 |
|  |  | US | 3 (23, 1043) | 0.024 (0.009, 0.063) | 73.82% |  |
|  |  | Germany | 1 (0, 45) | 0.011 (0.001, 0.151) | 0.00% |  |
|  |  | Italy | 2 (2, 810) | 0.003 (0.001, 0.011) | 0.00% |  |
|  |  | India | 3 (11, 144) | 0.082 (0.042, 0.152) | 20.33% |  |
|  |  | Iran | 3 (5, 206) | 0.037 (0.014, 0.093) | 9.82% |  |
|  |  | Poland | 2 (1, 128) | 0.026 (0.002, 0.305) | 60.13% |  |
|  |  | Algeria | 1 (0, 39) | 0.013 (0.001, 0.171) | 0.00% |  |
|  |  | Turkey | 2 (7, 93) | 0.076 (0.037, 0.150) | 0.00% |  |
|  |  | China | 5 (944, 1691) | 0.038 (0.001, 0.712) | 98.66% |  |
|  |  | Romania | 1 (0, 26) | 0.019 (0.001, 0.236) | 0.00% |  |
|  |  | Australia | 1 (0, 488) | 0.001 (0.000, 0.016) | 0.00% |  |
|  | who.regional.offices | western pacific region | 7 (944, 1766) | 0.029 (0.001, 0.492) | 98.20% | 0.935 |
|  |  | region of the americas | 3 (23, 1043) | 0.024 (0.009, 0.063) | 73.82% |  |
|  |  | european region | 10 (10, 1842) | 0.012 (0.004, 0.039) | 71.00% |  |
|  |  | south-east asia region | 3 (11, 144) | 0.082 (0.042, 0.152) | 20.33% |  |
|  |  | eastern mediterranean region | 3 (5, 206) | 0.037 (0.014, 0.093) | 9.82% |  |
|  |  | african region | 1 (0, 39) | 0.013 (0.001, 0.171) | 0.00% |  |
|  | ast_classification | CLSI | 23 (986, 4455) | 0.020 (0.004, 0.090) | 98.15% | 0.974 |
|  |  | Multiple Standards | 2 (2, 510) | 0.012 (0.000, 0.511) | 87.84% |  |
|  | infection.source | Bloodstream | 7 (8, 860) | 0.023 (0.006, 0.086) | 70.09% | 0.999 |
|  |  | Mixed | 14 (976, 3442) | 0.022 (0.003, 0.150) | 98.62% |  |
|  |  | urinary tract | 4 (3, 248) | 0.025 (0.006, 0.102) | 48.93% |  |
|  |  | Gastrointestinal tract | 1 (6, 450) | 0.013 (0.006, 0.029) | 0.00% |  |
|  | ast.methods | MIC based methods | 16 (44, 2845) | 0.025 (0.013, 0.045) | 67.11% | 0.623 |
|  |  | Disc Diffusion | 11 (949, 2195) | 0.028 (0.002, 0.262) | 97.69% |  |
| **moxifloxacin** | Overall | NA | 5 (87, 154) | 0.425 (0.106, 0.823) | 92.50% | NA |
| **nitrofurantoin** | Overall | NA | 11 (1232, 2598) | 0.385 (0.291, 0.490) | 94.50% | NA |
|  | year_group | 2000_2019 | 8 (699, 1425) | 0.354 (0.194, 0.555) | 94.56% | 0.681 |
|  |  | 2020_2024 | 3 (533, 1173) | 0.419 (0.285, 0.567) | 95.82% |  |
|  | country | India | 1 (36, 42) | 0.857 (0.717, 0.934) | 0.00% | <0.001 |
|  |  | Cuba | 1 (1, 10) | 0.100 (0.014, 0.467) | 0.00% |  |
|  |  | Iran | 4 (115, 518) | 0.212 (0.157, 0.280) | 62.00% |  |
|  |  | Algeria | 1 (23, 39) | 0.590 (0.432, 0.731) | 0.00% |  |
|  |  | US | 1 (1, 6) | 0.167 (0.023, 0.631) | 0.00% |  |
|  |  | China | 2 (828, 1495) | 0.554 (0.529, 0.579) | 0.00% |  |
|  |  | Australia | 1 (228, 488) | 0.467 (0.423, 0.512) | 0.00% |  |
|  | who.regional.offices | south-east asia region | 1 (36, 42) | 0.857 (0.717, 0.934) | 0.00% | <0.001 |
|  |  | region of the americas | 2 (2, 16) | 0.128 (0.032, 0.395) | 0.00% |  |
|  |  | eastern mediterranean region | 4 (115, 518) | 0.212 (0.157, 0.280) | 62.00% |  |
|  |  | african region | 1 (23, 39) | 0.590 (0.432, 0.731) | 0.00% |  |
|  |  | western pacific region | 2 (828, 1495) | 0.554 (0.529, 0.579) | 0.00% |  |
|  |  | european region | 1 (228, 488) | 0.467 (0.423, 0.512) | 0.00% |  |
|  | ast_classification | CLSI | 9 (981, 2071) | 0.346 (0.231, 0.484) | 95.54% | 0.472 |
|  |  | Multiple Standards | 1 (228, 488) | 0.467 (0.423, 0.512) | 0.00% |  |
|  | infection.source | Mixed | 7 (687, 1334) | 0.408 (0.234, 0.608) | 93.26% | 0.511 |
|  |  | urinary tract | 1 (12, 91) | 0.132 (0.076, 0.218) | 0.00% |  |
|  |  | Gastrointestinal tract | 2 (305, 685) | 0.393 (0.164, 0.681) | 97.90% |  |
|  |  | Bloodstream | 1 (228, 488) | 0.467 (0.423, 0.512) | 0.00% |  |
|  | ast.methods | Disc Diffusion | 7 (736, 1615) | 0.325 (0.176, 0.521) | 96.48% | 0.224 |
|  |  | MIC based methods | 4 (496, 983) | 0.510 (0.438, 0.581) | 65.09% |  |
| **penicillin** | Overall | NA | 16 (1353, 2358) | 0.719 (0.526, 0.855) | 97.14% | NA |
|  | year_group | 2000_2019 | 10 (972, 1368) | 0.733 (0.493, 0.886) | 88.73% | 0.843 |
|  |  | 2020_2024 | 6 (381, 990) | 0.703 (0.386, 0.899) | 97.74% |  |
|  | country | Taiwan | 2 (2, 75) | 0.035 (0.010, 0.112) | 0.00% | <0.001 |
|  |  | India | 3 (112, 125) | 0.942 (0.578, 0.995) | 83.27% |  |
|  |  | Turkey | 3 (117, 163) | 0.914 (0.178, 0.998) | 93.70% |  |
|  |  | US | 1 (32, 32) | 0.985 (0.799, 0.999) | 0.00% |  |
|  |  | Brazil | 1 (0, 4) | 0.100 (0.006, 0.674) | 0.00% |  |
|  |  | China | 3 (890, 1201) | 0.869 (0.655, 0.959) | 92.08% |  |
|  |  | Iran | 2 (143, 270) | 0.714 (0.235, 0.953) | 93.47% |  |
|  |  | Australia | 1 (57, 488) | 0.117 (0.091, 0.148) | 0.00% |  |
|  | who.regional.offices | western pacific region | 5 (892, 1276) | 0.540 (0.203, 0.843) | 94.28% | 0.460 |
|  |  | south-east Asia region | 3 (112, 125) | 0.942 (0.578, 0.995) | 83.27% |  |
|  |  | European region | 4 (174, 651) | 0.688 (0.275, 0.928) | 95.67% |  |
|  |  | region of the Americas | 2 (32, 36) | 0.732 (0.005, 0.999) | 89.52% |  |
|  |  | eastern Mediterranean region | 2 (143, 270) | 0.714 (0.235, 0.953) | 93.47% |  |
|  | ast_classification | CLSI | 15 (1296, 1870) | 0.758 (0.615, 0.859) | 92.99% | 0.005 |
|  |  | Multiple Standards | 1 (57, 488) | 0.117 (0.091, 0.148) | 0.00% |  |
|  | Infection source | Bloodstream | 5 (124, 628) | 0.403 (0.061, 0.875) | 90.52% | 0.002 |
|  |  | Mixed | 9 (1117, 1491) | 0.867 (0.742, 0.936) | 91.52% |  |
|  |  | Gastrointestinal tract | 2 (112, 239) | 0.349 (0.083, 0.762) | 49.39% |  |
|  | AST methods | MIC based methods | 5 (194, 674) | 0.782 (0.164, 0.985) | 94.84% | 0.459 |
|  |  | Disc Diffusion | 11 (1159, 1684) | 0.737 (0.588, 0.845) | 93.17% |  |
| **Quinupristin dalfopristin** | Overall | NA | 13 (514, 1397) | 0.241 (0.091, 0.502) | 97.09% | NA |
|  | year_group | 2000_2019 | 10 (487, 1281) | 0.231 (0.069, 0.548) | 97.68% | 0.854 |
|  |  | 2020_2024 | 3 (27, 116) | 0.282 (0.043, 0.774) | 91.50% |  |
|  | country | Taiwan | 2 (26, 75) | 0.489 (0.000, 1.000) | 95.00% | 0.001 |
|  |  | US | 3 (40, 435) | 0.068 (0.020, 0.202) | 86.27% |  |
|  |  | South Korea | 1 (269, 330) | 0.815 (0.770, 0.853) | 0.00% |  |
|  |  | Turkey | 2 (3, 93) | 0.033 (0.011, 0.097) | 0.00% |  |
|  |  | Iran | 3 (130, 150) | 0.810 (0.375, 0.968) | 84.17% |  |
|  |  | China | 1 (0, 62) | 0.008 (0.000, 0.115) | 0.00% |  |
|  | who.regional.offices | western pacific region | 4 (295, 467) | 0.311 (0.012, 0.943) | 94.00% | 0.015 |
|  |  | region of the Americas | 3 (40, 435) | 0.068 (0.020, 0.202) | 86.27% |  |
|  |  | European region | 3 (49, 345) | 0.073 (0.017, 0.268) | 79.28% |  |
|  |  | eastern Mediterranean region | 3 (130, 150) | 0.810 (0.375, 0.968) | 84.17% |  |
|  | ast_classification | CLSI | 12 (513, 1375) | 0.269 (0.100, 0.549) | 97.29% | 0.372 |
|  |  | Multiple Standards | 1 (1, 22) | 0.045 (0.006, 0.261) | 0.00% |  |
|  | infection.source | Bloodstream | 5 (304, 637) | 0.253 (0.017, 0.866) | 97.88% | >0.999 |
|  |  | Mixed | 6 (203, 728) | 0.228 (0.095, 0.456) | 91.91% |  |
|  |  | urinary tract | 2 (7, 32) | 0.227 (0.010, 0.895) | 87.70% |  |
|  | ast.methods | MIC based methods | 9 (365, 1170) | 0.125 (0.035, 0.360) | 97.85% | 0.029 |
|  |  | Disc Diffusion | 4 (149, 227) | 0.753 (0.088, 0.990) | 90.72% |  |
| **rifampicin** | Overall | NA | 11 (740, 2133) | 0.491 (0.315, 0.670) | 97.34% | NA |
|  | year_group | 2000_2019 | 8 (486, 1796) | 0.387 (0.250, 0.546) | 95.40% | 0.036 |
|  |  | 2020_2024 | 3 (254, 337) | 0.761 (0.254, 0.968) | 97.46% |  |
|  | country | Taiwan | 1 (5, 50) | 0.100 (0.042, 0.219) | 0.00% | 0.761 |
|  |  | Lebanon | 1 (20, 35) | 0.571 (0.406, 0.723) | 0.00% |  |
|  |  | Cuba | 1 (6, 10) | 0.600 (0.297, 0.842) | 0.00% |  |
|  |  | South Korea | 1 (122, 330) | 0.370 (0.319, 0.423) | 0.00% |  |
|  |  | Italy | 1 (96, 260) | 0.369 (0.313, 0.430) | 0.00% |  |
|  |  | Brazil | 1 (1, 4) | 0.250 (0.034, 0.762) | 0.00% |  |
|  |  | China | 2 (236, 1107) | 0.471 (0.052, 0.936) | 98.72% |  |
|  |  | Iran | 2 (236, 270) | 0.888 (0.782, 0.946) | 38.00% |  |
|  |  | Turkey | 1 (18, 67) | 0.269 (0.176, 0.387) | 0.00% |  |
|  | who.regional.offices | western pacific region | 4 (363, 1487) | 0.330 (0.156, 0.568) | 97.46% | 0.015 |
|  |  | eastern mediterranean region | 3 (256, 305) | 0.824 (0.568, 0.943) | 89.51% |  |
|  |  | region of the americas | 2 (7, 14) | 0.490 (0.200, 0.787) | 22.64% |  |
|  |  | european region | 2 (114, 327) | 0.331 (0.242, 0.434) | 57.40% |  |
|  | infection.source | Bloodstream | 2 (127, 380) | 0.214 (0.051, 0.580) | 91.50% | 0.146 |
|  |  | Mixed | 7 (409, 1514) | 0.527 (0.329, 0.717) | 96.17% |  |
|  |  | Gastrointestinal tract | 2 (204, 239) | 0.644 (0.094, 0.969) | 84.22% |  |
|  | ast.methods | MIC based methods | 3 (223, 640) | 0.305 (0.212, 0.417) | 83.40% | 0.146 |
|  |  | Disc Diffusion | 8 (517, 1493) | 0.597 (0.283, 0.847) | 98.05% |  |
| **streptomycin** | Overall | NA | 18 (1377, 2854) | 0.463 (0.361, 0.568) | 95.30% | NA |
|  | year_group | 2000_2019 | 11 (805, 1977) | 0.405 (0.313, 0.504) | 92.35% | 0.080 |
|  |  | 2020_2024 | 7 (572, 877) | 0.540 (0.334, 0.734) | 92.91% |  |
|  | country | Lebanon | 1 (9, 35) | 0.257 (0.140, 0.425) | 0.00% | 0.882 |
|  |  | Kuwait | 1 (76, 354) | 0.215 (0.175, 0.261) | 0.00% |  |
|  |  | Hong Kong | 1 (20, 35) | 0.571 (0.406, 0.723) | 0.00% |  |
|  |  | Turkey | 2 (72, 96) | 0.689 (0.229, 0.943) | 93.36% |  |
|  |  | Cuba | 1 (3, 10) | 0.300 (0.100, 0.624) | 0.00% |  |
|  |  | India | 1 (13, 33) | 0.394 (0.244, 0.566) | 0.00% |  |
|  |  | South Korea | 1 (106, 330) | 0.321 (0.273, 0.374) | 0.00% |  |
|  |  | Italy | 3 (478, 878) | 0.335 (0.132, 0.624) | 97.83% |  |
|  |  | US | 1 (303, 640) | 0.473 (0.435, 0.512) | 0.00% |  |
|  |  | Poland | 3 (108, 146) | 0.524 (0.077, 0.935) | 90.93% |  |
|  |  | Iran | 2 (29, 45) | 0.637 (0.469, 0.777) | 12.50% |  |
|  | who.regional.offices | eastern mediterranean region | 4 (114, 434) | 0.392 (0.183, 0.651) | 90.51% | 0.866 |
|  |  | western pacific region | 2 (126, 365) | 0.430 (0.216, 0.675) | 87.83% |  |
|  |  | european region | 9 (818, 1372) | 0.525 (0.373, 0.673) | 94.73% |  |
|  |  | region of the americas | 2 (306, 650) | 0.460 (0.369, 0.554) | 12.12% |  |
|  |  | south-east asia region | 1 (13, 33) | 0.394 (0.244, 0.566) | 0.00% |  |
|  | ast_classification | CLSI | 17 (1375, 2836) | 0.481 (0.377, 0.587) | 95.49% | 0.076 |
|  |  | EUCAST | 1 (2, 18) | 0.111 (0.028, 0.352) | 0.00% |  |
|  | infection.source | Mixed | 12 (1127, 2318) | 0.426 (0.309, 0.552) | 96.02% | 0.296 |
|  |  | Gastrointestinal tract | 1 (20, 35) | 0.571 (0.406, 0.723) | 0.00% |  |
|  |  | Bloodstream | 3 (121, 366) | 0.332 (0.283, 0.385) | 1.48% |  |
|  |  | urinary tract | 2 (109, 135) | 0.712 (0.344, 0.921) | 82.14% |  |
|  | ast.methods | Disc Diffusion | 10 (607, 1223) | 0.394 (0.213, 0.609) | 96.53% | 0.283 |
|  |  | MIC based methods | 8 (770, 1631) | 0.520 (0.412, 0.626) | 92.65% |  |
| **teicoplanin** | Overall | NA | 30 (1023, 4651) | 0.143 (0.086, 0.228) | 95.70% | NA |
|  | year_group | 2000_2019 | 22 (829, 3097) | 0.142 (0.079, 0.242) | 94.83% | 0.619 |
|  |  | 2020_2024 | 7 (194, 1518) | 0.179 (0.055, 0.452) | 96.62% |  |
|  | country | Taiwan | 2 (44, 75) | 0.563 (0.347, 0.757) | 69.26% | 0.681 |
|  |  | Lebanon | 1 (0, 35) | 0.014 (0.001, 0.187) | 0.00% |  |
|  |  | Kuwait | 1 (7, 354) | 0.020 (0.009, 0.041) | 0.00% |  |
|  |  | US | 2 (197, 403) | 0.304 (0.000, 1.000) | 97.74% |  |
|  |  | Germany | 1 (0, 45) | 0.011 (0.001, 0.151) | 0.00% |  |
|  |  | India | 5 (28, 237) | 0.066 (0.012, 0.282) | 88.22% |  |
|  |  | Turkey | 3 (70, 118) | 0.251 (0.001, 0.991) | 93.82% |  |
|  |  | Cuba | 1 (1, 10) | 0.100 (0.014, 0.467) | 0.00% |  |
|  |  | South Korea | 1 (5, 330) | 0.015 (0.006, 0.036) | 0.00% |  |
|  |  | Iran | 4 (118, 466) | 0.238 (0.041, 0.695) | 96.68% |  |
|  |  | Poland | 1 (0, 3) | 0.125 (0.007, 0.734) | 0.00% |  |
|  |  | Algeria | 1 (0, 39) | 0.013 (0.001, 0.171) | 0.00% |  |
|  |  | China | 2 (341, 1107) | 0.074 (0.001, 0.812) | 88.00% |  |
|  |  | Hungary | 1 (34, 71) | 0.479 (0.366, 0.594) | 0.00% |  |
|  |  | Italy | 2 (19, 618) | 0.031 (0.020, 0.049) | 0.00% |  |
|  |  | Australia | 1 (54, 488) | 0.111 (0.086, 0.142) | 0.00% |  |
|  | who.regional.offices | western pacific region | 5 (390, 1512) | 0.180 (0.054, 0.456) | 95.56% | 0.667 |
|  |  | eastern mediterranean region | 6 (125, 855) | 0.104 (0.016, 0.459) | 97.29% |  |
|  |  | region of the americas | 3 (198, 413) | 0.224 (0.001, 0.993) | 96.75% |  |
|  |  | european region | 10 (282, 1595) | 0.144 (0.054, 0.331) | 96.23% |  |
|  |  | south-east asia region | 5 (28, 237) | 0.066 (0.012, 0.282) | 88.22% |  |
|  |  | african region | 1 (0, 39) | 0.013 (0.001, 0.171) | 0.00% |  |
|  | ast_classification | CLSI | 25 (935, 3995) | 0.153 (0.086, 0.258) | 95.81% | 0.318 |
|  |  | Multiple Standards | 2 (54, 510) | 0.086 (0.027, 0.245) | 30.40% |  |
|  |  | EUCAST | 1 (34, 71) | 0.479 (0.366, 0.594) | 0.00% |  |
|  | infection.source | Bloodstream | 8 (300, 1190) | 0.202 (0.045, 0.576) | 96.79% | 0.683 |
|  |  | Mixed | 18 (675, 3042) | 0.124 (0.063, 0.227) | 95.66% |  |
|  |  | urinary tract | 2 (14, 113) | 0.092 (0.016, 0.391) | 51.81% |  |
|  |  | Gastrointestinal tract | 2 (34, 306) | 0.049 (0.000, 0.952) | 94.41% |  |
|  | ast.methods | MIC based methods | 12 (498, 1820) | 0.210 (0.082, 0.440) | 96.53% | 0.158 |
|  |  | Disc Diffusion | 18 (525, 2831) | 0.098 (0.047, 0.194) | 95.24% |  |
| **tetracycline** | Overall | NA | 24 (1935, 3534) | 0.511 (0.413, 0.609) | 94.80% | NA |
|  | year_group | 2000_2019 | 16 (1634, 2465) | 0.582 (0.478, 0.679) | 93.58% | 0.016 |
|  |  | 2020_2024 | 7 (275, 1033) | 0.321 (0.143, 0.573) | 96.06% |  |
|  | country | Lebanon | 1 (12, 35) | 0.343 (0.206, 0.512) | 0.00% | 0.003 |
|  |  | Kuwait | 1 (229, 354) | 0.647 (0.596, 0.695) | 0.00% |  |
|  |  | Hong Kong | 1 (19, 35) | 0.543 (0.379, 0.698) | 0.00% |  |
|  |  | Cuba | 1 (9, 10) | 0.900 (0.533, 0.986) | 0.00% |  |
|  |  | Taiwan | 1 (22, 25) | 0.880 (0.687, 0.961) | 0.00% |  |
|  |  | South Korea | 2 (289, 493) | 0.468 (0.046, 0.941) | 99.28% |  |
|  |  | Iran | 5 (331, 528) | 0.626 (0.579, 0.671) | 11.61% |  |
|  |  | Brazil | 1 (2, 4) | 0.500 (0.123, 0.877) | 0.00% |  |
|  |  | India | 2 (58, 94) | 0.631 (0.452, 0.780) | 62.78% |  |
|  |  | Algeria | 1 (27, 39) | 0.692 (0.533, 0.816) | 0.00% |  |
|  |  | Turkey | 3 (67, 160) | 0.309 (0.069, 0.727) | 94.67% |  |
|  |  | China | 3 (858, 1201) | 0.576 (0.340, 0.782) | 95.95% |  |
|  |  | Italy | 1 (9, 68) | 0.132 (0.070, 0.235) | 0.00% |  |
|  |  | Australia | 1 (3, 488) | 0.006 (0.002, 0.019) | 0.00% |  |
|  | who.regional.offices | eastern Mediterranean region | 7 (572, 917) | 0.613 (0.550, 0.672) | 62.19% | 0.001 |
|  |  | western pacific region | 7 (1188, 1754) | 0.587 (0.392, 0.758) | 97.10% |  |
|  |  | region of the Americas | 2 (11, 14) | 0.745 (0.254, 0.962) | 56.27% |  |
|  |  | south-east Asia region | 2 (58, 94) | 0.631 (0.452, 0.780) | 62.78% |  |
|  |  | African region | 1 (27, 39) | 0.692 (0.533, 0.816) | 0.00% |  |
|  |  | European region | 5 (79, 716) | 0.133 (0.026, 0.470) | 96.39% |  |
|  | ast_classification | CLSI | 20 (1877, 2949) | 0.562 (0.466, 0.653) | 94.24% | <0.001 |
|  |  | Multiple Standards | 2 (5, 510) | 0.023 (0.002, 0.268) | 88.57% |  |
|  | infection.source | Mixed | 12 (1298, 1979) | 0.542 (0.426, 0.654) | 93.61% | 0.688 |
|  |  | Gastrointestinal tract | 3 (167, 274) | 0.609 (0.550, 0.665) | 0.00% |  |
|  |  | Bloodstream | 5 (345, 937) | 0.482 (0.164, 0.814) | 96.87% |  |
|  |  | urinary tract | 4 (125, 344) | 0.381 (0.121, 0.733) | 95.43% |  |
|  | ast.methods | Disc Diffusion | 14 (1478, 2282) | 0.549 (0.448, 0.646) | 92.83% | 0.328 |
|  |  | MIC based methods | 10 (457, 1252) | 0.440 (0.227, 0.679) | 96.45% |  |
| **tigecycline** | Overall | NA | 7 (3, 1380) | 0.005 (0.002, 0.012) | 0.00% | NA |
| **Trimethoprim sulfamethaxazole** | Overall | NA | 8 (378, 666) | 0.618 (0.406, 0.793) | 92.81% | NA |
| **Erythromycin** | Overall | NA | 28 (3040, 4784) | 0.628 (0.545, 0.704) | 95.84% | NA |
|  | year_group | 2000_2019 | 18 (1944, 2814) | 0.666 (0.575, 0.746) | 93.69% | 0.215 |
|  |  | 2020_2024 | 10 (1096, 1970) | 0.554 (0.394, 0.703) | 97.14% |  |
|  | country | Lebanon | 1 (66, 111) | 0.595 (0.501, 0.682) | 0.00% | <0.001 |
|  |  | Kuwait | 1 (16, 32) | 0.500 (0.333, 0.667) | 0.00% |  |
|  |  | US | 1 (33, 38) | 0.868 (0.720, 0.944) | 0.00% |  |
|  |  | Germany | 1 (46, 103) | 0.447 (0.354, 0.543) | 0.00% |  |
|  |  | Cuba | 1 (44, 84) | 0.524 (0.418, 0.628) | 0.00% |  |
|  |  | India | 3 (101, 130) | 0.768 (0.674, 0.842) | 15.16% |  |
|  |  | South Korea | 1 (227, 249) | 0.912 (0.869, 0.941) | 0.00% |  |
|  |  | Italy | 2 (76, 97) | 0.554 (0.038, 0.975) | 94.30% |  |
|  |  | Iran | 5 (486, 912) | 0.512 (0.398, 0.625) | 88.48% |  |
|  |  | Brazil | 1 (9, 18) | 0.500 (0.284, 0.716) | 0.00% |  |
|  |  | Nigeria | 1 (2, 6) | 0.333 (0.084, 0.732) | 0.00% |  |
|  |  | Algeria | 1 (69, 80) | 0.863 (0.769, 0.922) | 0.00% |  |
|  |  | China | 5 (1467, 1995) | 0.691 (0.606, 0.764) | 90.49% |  |
|  |  | Uganda | 1 (2, 5) | 0.400 (0.100, 0.800) | 0.00% |  |
|  |  | Turkey | 1 (153, 175) | 0.874 (0.816, 0.916) | 0.00% |  |
|  |  | Egypt | 1 (33, 82) | 0.402 (0.302, 0.512) | 0.00% |  |
|  |  | Australia | 1 (210, 667) | 0.315 (0.281, 0.351) | 0.00% |  |
|  | continent | Asia | 17 (2516, 3604) | 0.666 (0.587, 0.736) | 94.23% | 0.348 |
|  |  | Americas | 3 (86, 140) | 0.650 (0.388, 0.844) | 83.56% |  |
|  |  | Europe | 3 (122, 200) | 0.532 (0.166, 0.867) | 94.67% |  |
|  |  | Africa | 4 (106, 173) | 0.543 (0.216, 0.838) | 91.12% |  |
|  |  | Oceania | 1 (210, 667) | 0.315 (0.281, 0.351) | 0.00% |  |
|  | who.regional.offices | eastern Mediterranean region | 6 (499, 956) | 0.507 (0.423, 0.592) | 80.16% | 0.280 |
|  |  | region of the Americas | 3 (86, 140) | 0.650 (0.388, 0.844) | 83.56% |  |
|  |  | European region | 5 (485, 1042) | 0.579 (0.280, 0.829) | 97.76% |  |
|  |  | south-east Asia region | 3 (101, 130) | 0.768 (0.674, 0.842) | 15.16% |  |
|  |  | western pacific region | 6 (1694, 2244) | 0.737 (0.645, 0.812) | 93.39% |  |
|  |  | African region | 3 (73, 91) | 0.595 (0.184, 0.906) | 82.61% |  |
|  | ast_classification | CLSI | 26 (2761, 4037) | 0.633 (0.561, 0.700) | 93.21% | 0.044 |
|  |  | Multiple Standards | 1 (210, 667) | 0.315 (0.281, 0.351) | 0.00% |  |
|  | infection.source | Mixed | 18 (1927, 2761) | 0.632 (0.546, 0.711) | 92.89% | 0.645 |
|  |  | Bloodstream | 5 (503, 1000) | 0.728 (0.334, 0.935) | 98.07% |  |
|  |  | urinary tract | 1 (279, 527) | 0.529 (0.487, 0.572) | 0.00% |  |
|  |  | Gastrointestinal tract | 3 (329, 491) | 0.530 (0.258, 0.785) | 93.55% |  |
|  | ast.methods | Disc Diffusion | 18 (1877, 2882) | 0.566 (0.478, 0.649) | 93.03% | 0.036 |
|  |  | MIC based methods | 10 (1163, 1902) | 0.738 (0.562, 0.861) | 97.70% |  |

**Figures**. Subgroup analyses of antibiotic resistance proportion based on AST, who regions, countries and infection source.


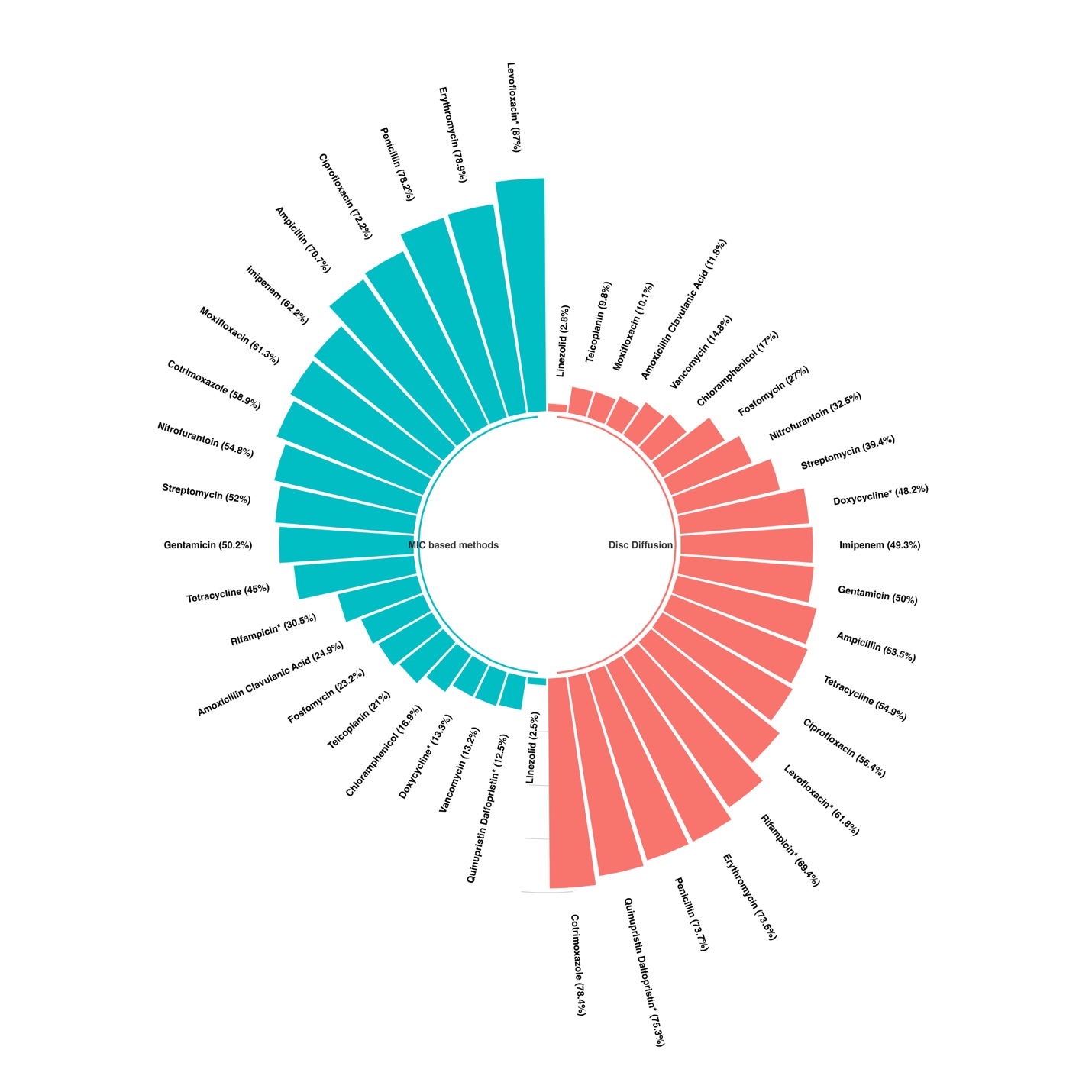

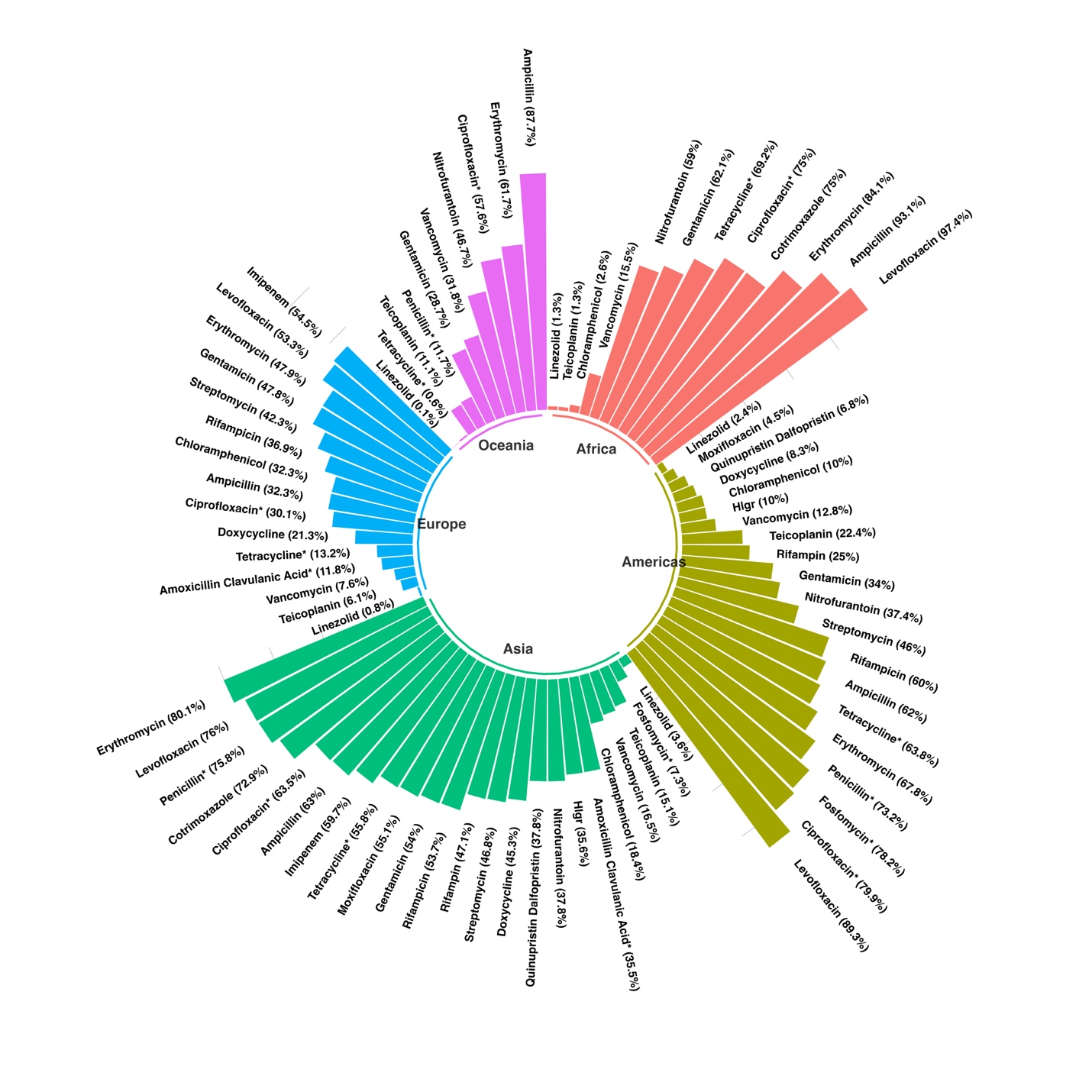

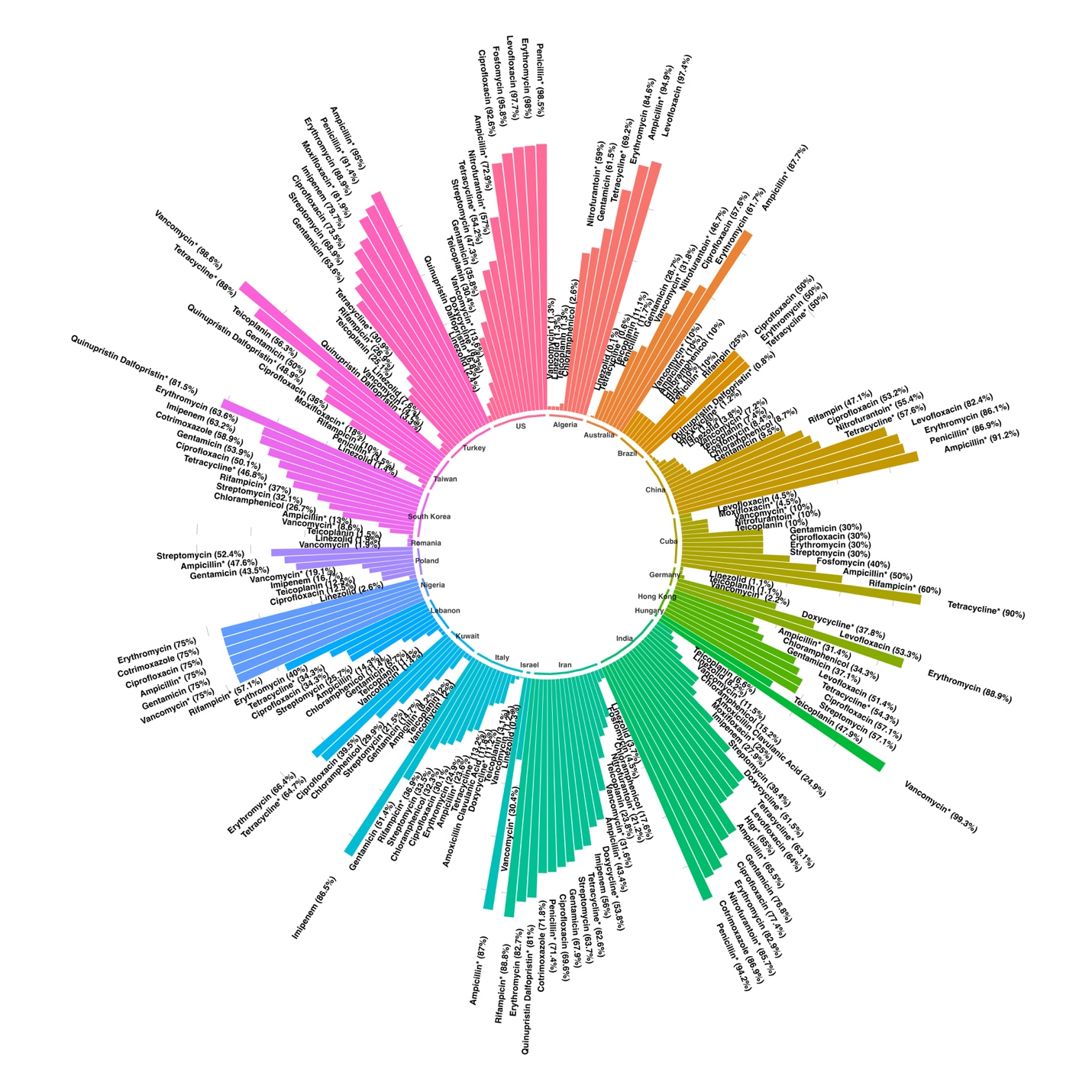

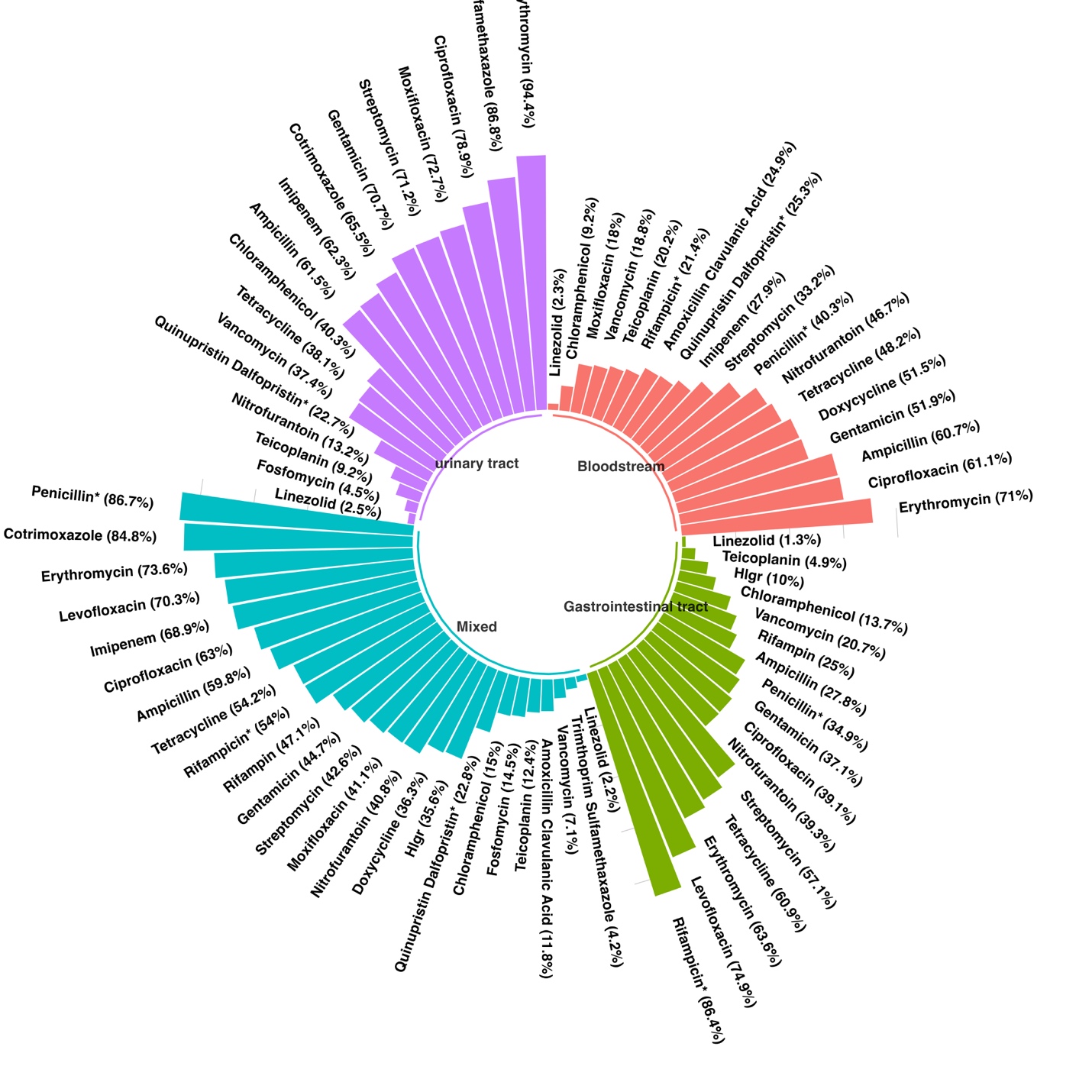

Supplement: Supplementary file 1 [file DataSheet1.docx]
